# Uncovering structural themes across cilia microtubule inner proteins with implications for human cilia function

**DOI:** 10.1038/s41467-024-46737-3

**Published:** 2024-03-27

**Authors:** Jens S. Andersen, Aaran Vijayakumaran, Christopher Godbehere, Esben Lorentzen, Vito Mennella, Kenneth Bødtker Schou

**Affiliations:** 1 Department of Biochemistry and Molecular Biology, University of Southern Denmark, Campusvej 55, 5230 Odense M, Denmark; 2 MRC Toxicology Unit, School of Biological Sciences, University of Cambridge, Gleeson Building, Tennis Court Road, CB2 1QR Cambridge, UK; 3 Department of Molecular Biology and Genetics, Aarhus University, Universitetsbyen 81, 8000 Aarhus C, Denmark; 4 Department of Pathology, School of Biological Sciences, University of Cambridge, 10 Tennis Court Road, CB2 1QP Cambridge, UK; 5 The Danish Cancer Society Research Center, Danish Cancer Institute, Strandboulevarden 49, 2100 Copenhagen, Denmark

**Keywords:** Cilia, Protein structure predictions, Microtubules, Computational biology and bioinformatics

## Abstract

Centrosomes and cilia are microtubule-based superstructures vital for cell division, signaling, and motility. The once thought hollow lumen of their microtubule core structures was recently found to hold a rich meshwork of microtubule inner proteins (MIPs). To address the outstanding question of how distinct MIPs evolved to recognize microtubule inner surfaces, we applied computational sequence analyses, structure predictions, and experimental validation to uncover evolutionarily conserved microtubule- and MIP-binding modules named NWE, SNYG, and ELLEn, and PYG and GFG-repeat by their signature motifs. These modules intermix with MT-binding DM10-modules and Mn-repeats in 24 *Chlamydomonas* and 33 human proteins. The modules molecular characteristics provided keys to identify elusive cross-species homologs, hitherto unknown human MIP candidates, and functional properties for seven protein subfamilies, including the microtubule seam-binding NWE and ELLEn families. Our work defines structural innovations that underpin centriole and axoneme assembly and demonstrates that MIPs co-evolved with centrosomes and cilia.

## Abstract (web summary)

The inside surface of microtubules contains so-called microtubule inner proteins, but little is known about their identity. Here the authors use bioinformatics to identify structural motifs within this class of proteins and potential new members.

## Introduction

An unexpected finding in recent cilia research is the discovery of a rich meshwork of proteins in the microtubule inner lumen^1–4^. Evidence obtained from motile cilia of various species^1,5–10^, indicates that the MT inner lumen of the centriole triplet, the axonemal outer doublet, and the central pair are decorated periodically with a meshwork of MT inner proteins (MIPs)^1,2^. Despite this great number of MIPs identified in the axonemal outer doublets of motile cilia, 35 found in *C. reinhardtii*, 29 in mammals, and nine MIPs recently found in the central apparatus of *C. reinhardtii*^4^, their function and molecular determinants of selectivity for the cilia MT lumen remain to be fully explored.

The structural properties of many MIPs do not appear to resemble those of any known proteins populating cilia or other cellular compartments. Most MIPs lack apparent catalytic activity, which has led to the hypothesis that they function as specialized adaptors or scaffolds. For MIPs extending to the MT outer wall, it has been proposed that they play a role in anterograde and retrograde IFT transport^1,2,11^. Other MIPs might serve to stabilize the axonemal outer doublets as recently suggested for a subset of central apparatus MIPs^4^, a role that seems plausible considering the mechanical stress imposed on the MT lattice as cilia bend. Intriguingly, some MIPs appear to localize specifically to the MT seam closing the A-tubule of the axonemal outer doublet, an unusual site among inter-protofilament interactions as it features heterotypic tubulin contacts and therefore represents a unique site on the MT^1^. In addition, the physical link between the seam of the A-tubule and the outer junction suggests that the location of the seam determines the site of B-tubule nucleation. Yet, no protein structure comprising a seam-reader module is known to target these MIPs specifically to the seam region of the A-tubule^1,2,12^. Despite these recent advances in our understanding of the motile cilia substructures and protein complexes in protist and mammalian systems, our knowledge of the structural and evolutionary relationships across MIPs and their structural relationship to other MT-associated proteins inside and outside cilia, if any, is less well established. Given the adeptness of recent computational algorithms in dissecting remote homologies of proteins, especially by means of the highly sensitive profile-HMM searches, here we resorted to a first of its kind exhaustive computational analysis of the full repertoire of MIP components uncovered in the axonemal outer doublet lumen across species. This analysis detects hitherto unknown protein modules and repeats in MIPs that collectively comprise seven evolutionary conserved protein families across eukaryotes. Besides the reported DM10 domain and Mn repeats, we define these microtubule inner surface binding-modules and repeats as the NWE, SNYG, and ELLEn modules, the PYG and GFG repeats, and the non-repeat Mn units. We validate hitherto unknown MIP candidates bearing these structural elements such as the DM10-containing CAPS2, the NWE-containing C11ORF1, the SNYG-bearing C2ORF50, as well as the ELLEn family member trichoplein (TCHP) and provide evidence for their functions in cilia. We discuss how the exclusive presence of these modules in MIPs facilitated the identification and functional assignment of novel MIPs across species.

## Results

### Overview of MIP architectural landscape analyses

**Fig. 1:**
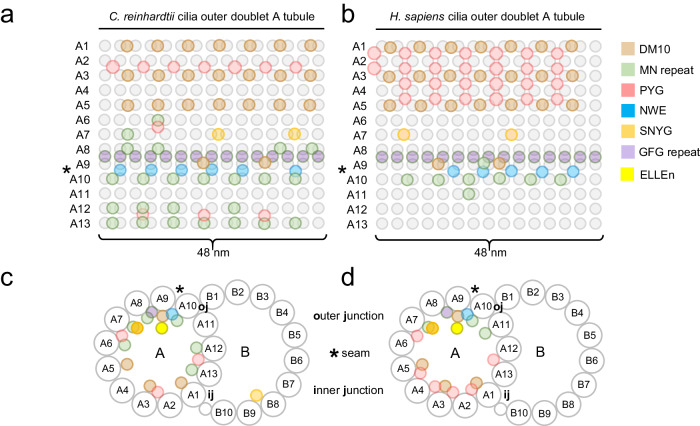
Schematic representation of the positions of MIP modules inside the motile cilia outer doublet of *C. reinhardtii* and *H. sapiens* based on cryo-EM structures of ciliary doublet microtubules. **a**, **b** Distribution of MIPs along the axoneme at periodically defined positions. **c**, **d** Lateral distribution of MIPs at the protofilaments of the A- and B-tubules. The position of the seam and of the outer and inner junctions between the A- and B-tubules are indicated.

**Fig. 2:**
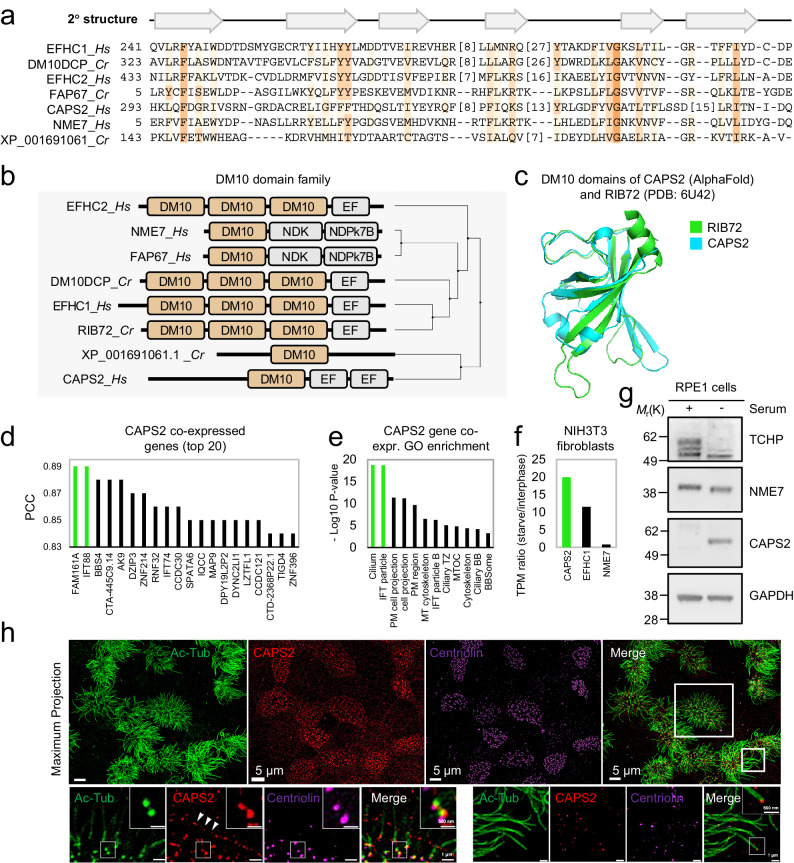
New members of the DM10 domain containing protein family. **a** Multiple sequence alignment of the DM10 domain from the human and *C. reinhardtii* proteins identified by iterative HMM-to-HMM searches. Proteins are designated by their UniProt identifiers. Coloring schemes as per ClustalX parameters with modifications. Predicted regions corresponding to β-sheets and based on RIB72 DM10 domain, are shown above the alignment. **b** Schematic representation of protein architectures of the DM10 domain family. Phylogenetic relationships were calculated using average distances and percent identity (PID) between DM10 modules used in the alignment. **c** Superimposition of *C. reinhardtii* RIB72 DM10 domain structure with the AlphaFold2-predicted DM10 domain structure of CAPS2. **d** Gene co-expression analysis of CAPS2 across non-cancerous ciliated Head and Neck Squamous cell Carcinoma (HNSC), Kidney renal clear cell carcinoma (KICH), Lung Adenocarcinoma (LUAD), Lung Squamous cell Carcinoma (LUSC), and Uterine Corpus Endometrial Carcinoma (UCEC) tissues. PCC = Pearson correlation coefficient. **e** Gene ontology (GO) enrichment analysis of CAPS2 co-expressed genes identified in (**d**). Enrichment *P*-values were obtained using the PANTHER Classification System for the slim cellular component devised by the PANTHER software^57^. **f** RNA-seq ratios obtained from NIH3T3 fibroblast extracts. Ratios calculated as transcripts per million (TPM) of serum starved cells (24 h) over cycling cells. Source data are provided as a Source Data file. **g** Immunoblot of cell extracts from hTERT-RPE1 cells grown with or without serum. Proteins were probed with indicated antibodies. The shown immunoblot is representative of two independent experiments. **h** Immunofluorescence microscopy micrographs of motile cilia on human bronchial epithelial cells imaged by structured illumination microscopy. Cells were probed with the indicated antibodies. The images are representative of two independent experiments. Source data are provided as a Source Data file. Scale bar: 5 μm in the top row, 1 μm in the second row.

**Fig. 3:**
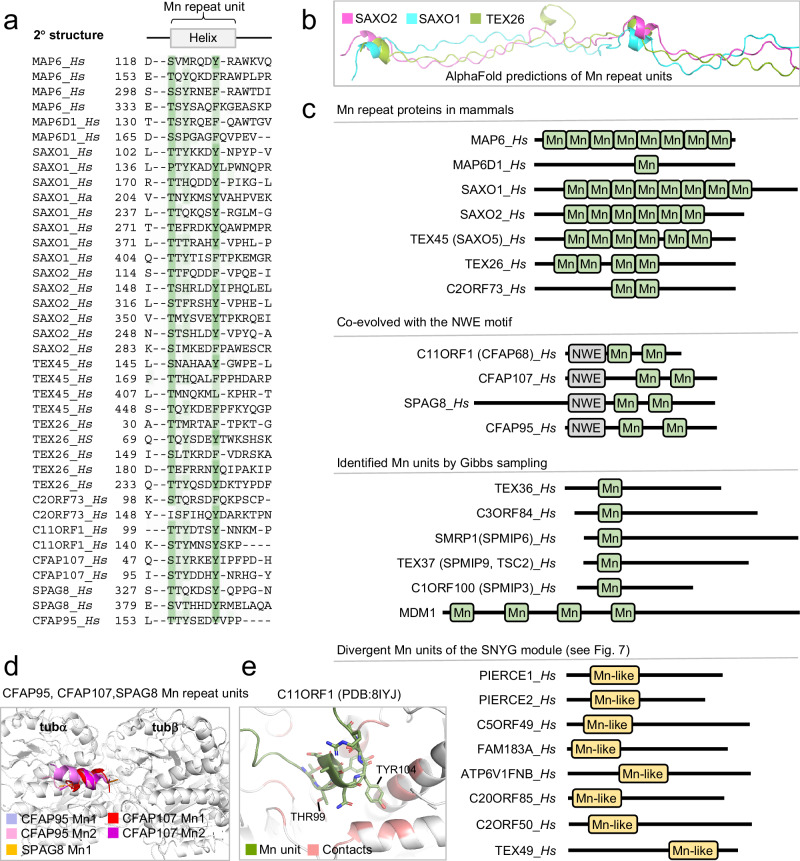
Identification of Mn repeats in eukaryotic MIPs. **a** Multiple sequence alignment of the Mn repeat units from human proteins identified by iterative HMM-to-HMM searches. The expanded alignment with *C. reinhardtii* Mn repeat proteins is shown in supplementary Fig. S2c. Proteins are designated by their UniProt identifiers. Coloring schemes as per ClustalX parameters with modifications. Shown in green are positions where hydrophilicity and aromatic residues are conserved and participate in the short α-helix of individual Mn repeat unit. Predicted regions corresponding to α-helices and based on FAP363 Mn repeats, are shown above the alignment. **b** Portions of the elongated Mn repeat proteins SAXO1, SAXO2, and TEX26 as predicted by comparative modeling as well as deep learning by AlphaFold2. **c** Module architecture of proteins with known and predicted Mn repeats and inferred Mn repeat. **d** Superimposed Mn repeat units of human CFAP95, CFAP107, and SPAG8as assessed by Pymol. **e** Examples of Mn repeat unit contact sites with the MT lattice in mammals. The Mn unit is shown in green, and MT contact side chains are shown in red.

**Fig. 4:**
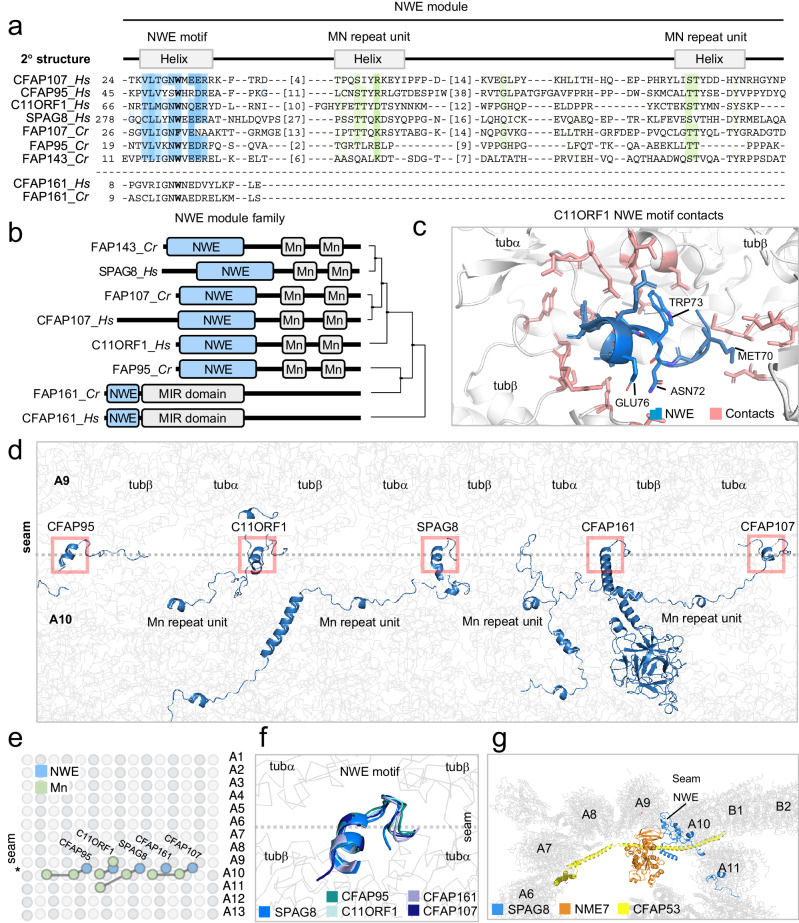
Identification of a universal seam-binding module in MIPs across eukaryotes. **a** Multiple sequence alignment of the NWE module from the five human and four *C. reinhardtii* proteins identified by iterative HMM-to-HMM searches. Except for CFAP161, the NWE module consists of the co-evolved triad of an N-terminal NWE motif adjoined to two Mn repeat units. In FAP161 and CFAP161 the Mn repeats are absent. The amino acid numbers of the Mn repeat units are indicated. Coloring schemes as per ClustalX parameters with modifications. Positions comprising the NWE motif are highlighted in blue. The Mn repeat units are colored green as in Fig. 3. Predicted regions corresponding to α-helices are shown above the alignment. **b** Module architecture of predicted NWE module proteins in humans and *C. reinhardtii*. Phylogenetic relationships were calculated using average distances and percent identity (PID) between NWE modules used in the alignment. **c** The NWE motif in C11ORF1 as found in complex with the MTs inner surface MTs in the *H. sapiens* cilia outer doublet. **d** NWE motifs and Mn repeats across the cilia outer doublet seam in *H. sapiens*. **e** Distribution of the NWE motifs and Mn repeats along the axoneme at periodically defined positions. **f** Structural superimposition of the NWE motifs at the seam. **g** The NWE motif of SPAG8 binds MTs at the A-tubule seam region of the outer doublet. NME7 associates with the ELLEn module of CFAP53 (see Fig. 8).

**Fig. 5:**
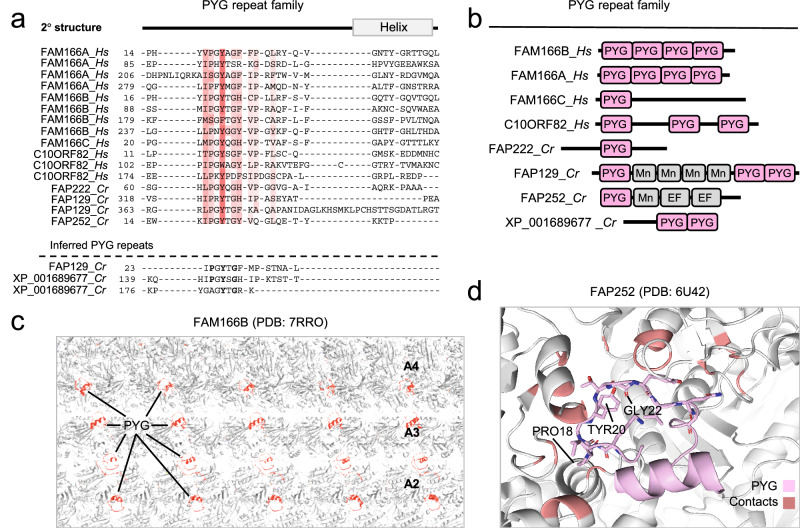
Identification of MT inner surface binding PYG repeats in MIPs. **a** Multiple sequence alignment of the PYG repeats in four human and three *C. reinhardtii* proteins identified by HMM-to-HMM searches. Only the outermost N-terminal PYG repeat unit for each protein is shown. Proteins are designated by their UniProt identifiers. Coloring schemes as per ClustalW parameters with modifications. **b** Module architecture of predicted PYG repeat proteins in human and *C. reinhardtii*. **c** PYG repeats of human FAM166B shown in the outer doublet lumen MT interface. **d** Structure of the PYG repeat in FAP252 where contacts between the MT lattice and the PYG repeat is shown for the conserved residues.

**Fig. 6:**
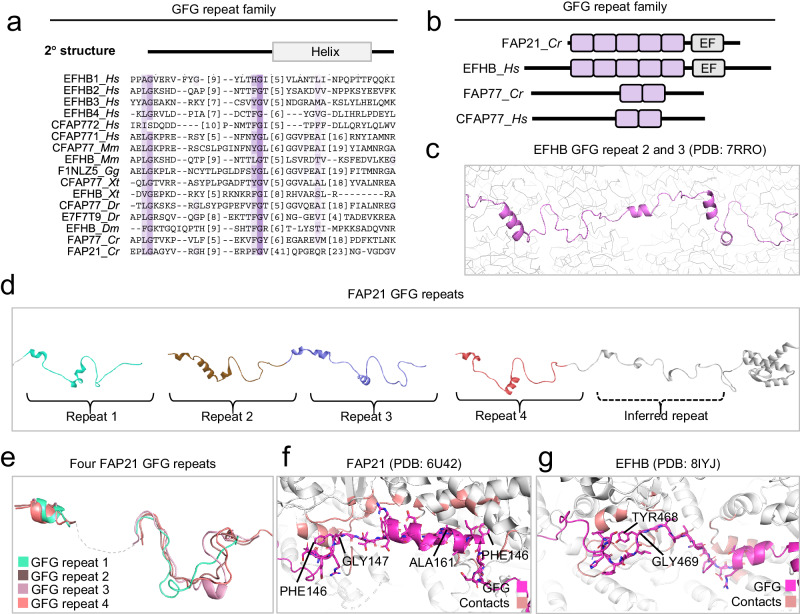
Identification of MT inner surface binding GFG repeats in MIPs. **a** Multiple sequence alignment of the GFG repeat in two human and two *C. reinhardtii* proteins identified by iterative HMM-to-HMM searches. Proteins are designated by their UniProt identifiers. Coloring schemes as per ClustalW parameters with modifications. **b** Module architecture and evolutionary relationships of the GFG repeat family members in *C. reinhardtii* and humans. **c** The GFG repeat 2 in human EFHB. **d** Module architecture of *C. reinhardtii* FAP21 (human EFHB) showing the GFG repeats along the length of FAP21. In FAP21, four GFG repeats were identified by sequence alignment and a fifth was inferred from visual inspection of the FAP21 tertiary structure in the *C. reinhardtii* outer doublet. **e** Superimposition of four GFG repeats from FAP21. The highly divergent central portion of each GFG repeat (dotted line) is omitted to allow superimposition. **f** One GFG repeat unit of FAP21 in contact with the MT lattice. Contact sites are highlighted in red. Conserved aromatic and glycine residues are shown (**g**) One GFG repeat unit of EFHB in contact with the MT lattice. Contact sites are highlighted in red. Conserved aromatic and glycine residues are shown.

**Fig. 7:**
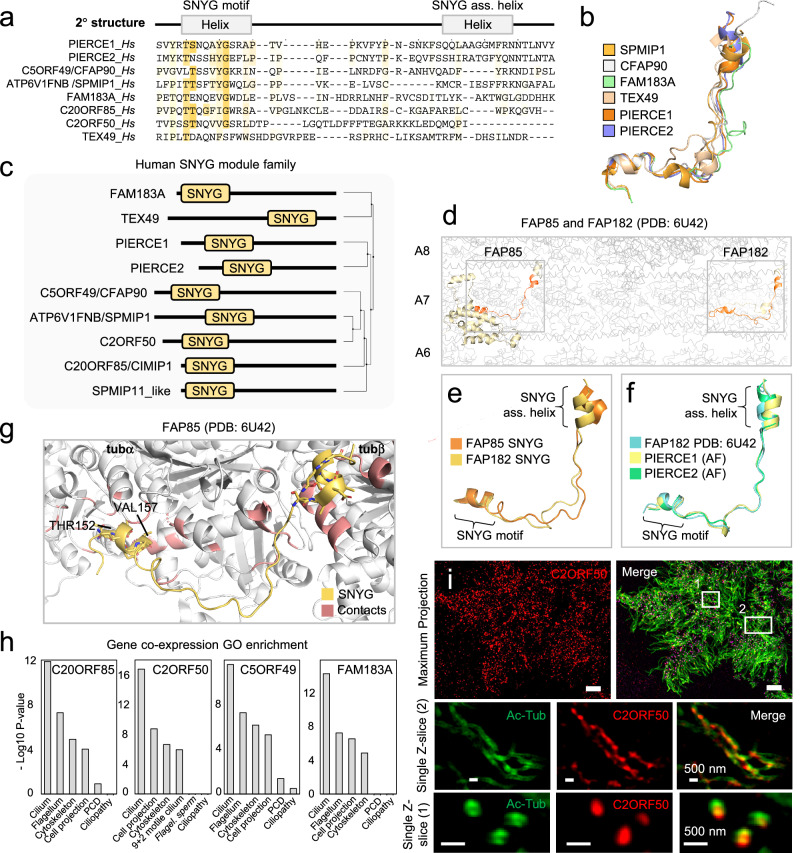
Identification of SNYG modules in *C. reinhardtii* and mammalian MIPs. **a** Multiple sequence alignment of the SNYG module in eight human proteins identified by iterative HMM-to-HMM searches. The SNYG module consists of two α-helices, one representing the conserved Mn repeat-like motif bearing a highly conserved glycine, and the other helix here named the SNYG motif associated helix (SNYG ass. helix). Proteins are designated by their UniProt identifiers. Coloring schemes as per ClustalW parameters with modifications. Predicted regions corresponding to α-helices are shown above the alignment. **b** Superimposition of experimentally determined SNYG modules from mammalian PIERCE1, PIERCE2, TEX49, FAM183A, C5ORF49 (CFAP90), and ATP6V1FNB (SPMIP1). Structures were derived from PDB: 8OTZ. **c** Module architecture of predicted SNYG module proteins in human. **d** The SNYG module in FAP85 and FAP182 (shown in boxes) in complex with the tubulin lattice of the *C. reinhardtii* cilia outer doublet A-tubule (PDB: 6U42). Protofilament number shown right. **e** Superimposition of SNYG module structures from FAP85 and FAP182. **f** Superimpositions of FAP182 SNYG module structures with AlphaFold2-predicted SNYG modules of PIERCE1 and PIERCE2. **g** The FAP85 SNYG modules in complex with tubA and tubB. Contact sites between tubulins and conserved threonine or hydrophobic residues are highlighted in red. **h** GO enrichment of gene co-expression analysis (top 6) of the four SNYG family members C20ORF85, C2ORF50, C5ORF49, and FAM183A. Enrichment *P*-values were obtained using the PANTHER Classification System for the slim cellular component devised by the PANTHER software^57^. **i** Immunofluorescence microscopy micrographs of motile cilia on cultured human bronchial epithelial cells imaged by structured illumination microscopy. Cells were stained with indicated antibodies. The images are representative of two independent experiments. Scale bar: 5 μm in the top row, 500 nm in the second row.

**Fig. 8:**
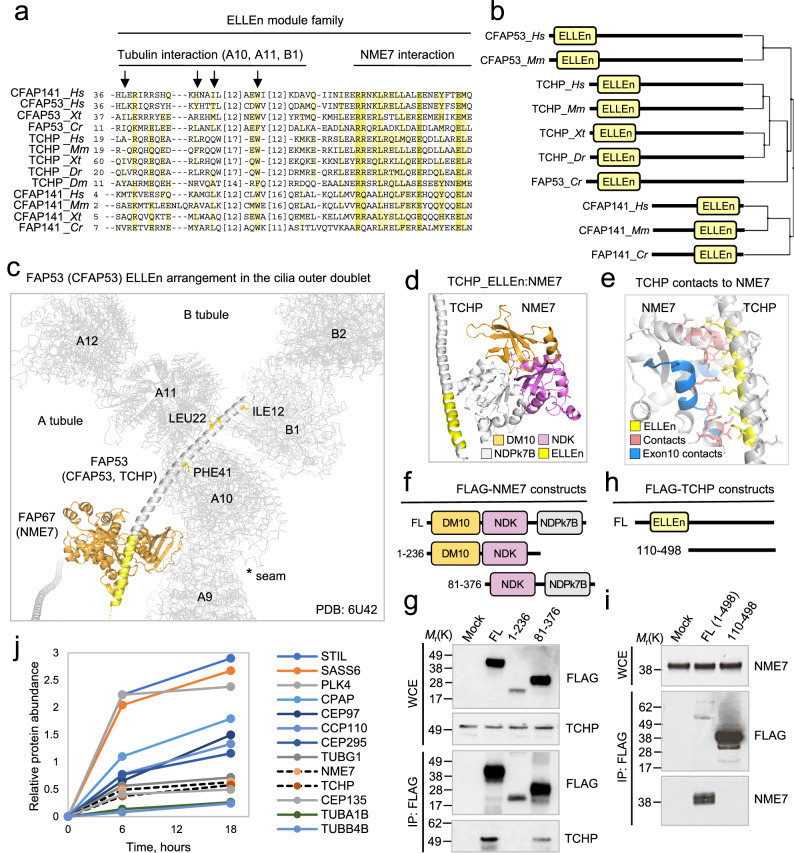
The ELLEn module binds FAP67 and NME7 in the cilia outer doublet lumen. **a** Multiple sequence alignment of the ELLEn module across species. Proteins are designated by their UniProt identifiers. Species represented are Homo sapiens (Hs), *Mus musculus* (Mm), *Xenopus tropicalis* (Xt), *Danio rerio* (Dr), *Drosophila melanogaster* (Dm), and *C. reinhardtii* (Cr). Coloring schemes as per ClustalW parameters with modifications. **b** Module architecture and evolutionary relationships of the ELLEn module family members across species. Phylogenetic relationships are calculated using average distances and percent identity (PID) between ELLEn modules used in the alignment. **c** Associations of the ELLEn module in FAP53 with FAP67 (NME7 in human). **d**, **e** AlphaFold2 prediction of the human complex and the contacts between the ELLEn module of TCHP and NME7. **f** Schematic representations of FLAG-NME7 constructs. **g** FLAG pulldown analysis and immunoblot of FLAG-NME7 constructs expressed in HEK293T cells. Proteins were probed with the indicated antibodies. The immunoblots are representative of two independent experiments. **h** Schematic representations of FLAG-TCHP constructs. **i** FLAG pulldown analysis and immunoblot of FLAG-TCHP constructs expressed in HEK293T cells. Proteins were probed with the indicated antibodies. The immunoblots are representative of three independent experiments. **j** Quantitative analysis of centrosome-associated proteins during centriole biogenesis. An array of representative centrosome proteins implicated in centriole duplication are shown in solid lines indicating temporal dynamics of centrosome protein recruitments. NME7 and TCHP are indicated with dashed lines. The quantitative analysis is representative of experiments performed in two replicates. Source data are provided as a Source Data file.

**Fig. 9:**
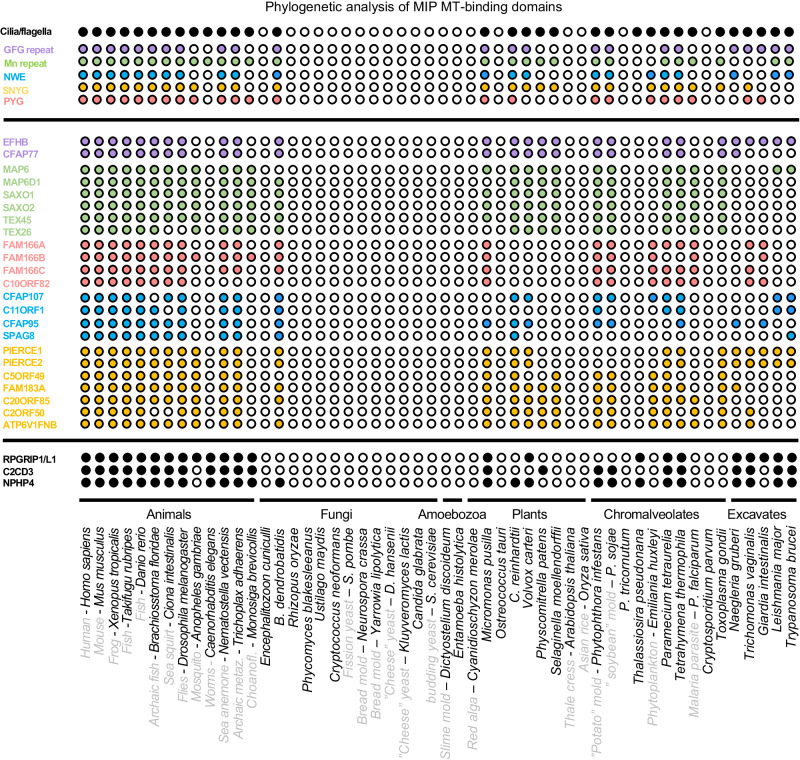
Co-evolution of MIP modules with centrosomes and cilia. Coulson plot demonstrating occurrence and absence (or loss) of identified MIP modules in 52 eukaryotic genomes. Rows show individual proteins or groups of proteins divided into the five module families GFG repeat (purple), Mn repeat (green), NWE module (blue), SNYG module (yellow), and PYG repeat (pink). The presence of a centrosome or cilium is shown for each organism in the top row.

Given the unclear phylogeny of MIPs across eukaryotes, we set out to answer three questions concerning MIPs in cilia architectures and their evolution. First, are the seemingly disparate array of MIPs evolutionary and structurally related, and specifically, do MIPs share structural modules allowing inner surface interactions within the cilia outer doublet and central pair MTs? Second, if indeed MIPs bear specialized MT inner surface binding-modules, how might these contribute to the formation of the cilia outer doublet architecture? Third, how are MIPs specifically confined to the MT lumen of cilia outer doublets? Currently, the experimentally determined structures of MIPs across eukaryotes reveal little as to how such a variety of protein structures evolved their preference for the cilia MT inner interface niche. We therefore assessed the structural properties and relationships among MIPs across eukaryotes by computational sequence analysis and remote homology detection methods. Iterative sequence-to-profile BLAST searches against a non-redundant sequence database, however, uncovered no significant matches to other proteins besides closely related FAP homologs in *C. reinhardtii* (not shown). Hence, we explored their possible structural relationships in protists and humans by systematically assessing their sequence homologies to the protein profiles present in the PFAM database using the more sensitive hidden Markov-based profile-to-profile (profile-HMM) searches. The result of this survey is summarized in Table 1 and Fig. 1 and discussed in additional detail in the following sections. Collectively, we revealed seven evolutionary conserved protein families across eukaryotes based on homologies to the DM10 domain (Fig. 2), the Mn repeats (Fig. 3), a hitherto unknown MT seam-binding module defined in this study named NWE (Fig. 4), two MT-binding protein repeat families, namely PYG and GFG repeats (Figs. 5 and 6), and a MT-binding module also defined in this study named SNYG (Fig. 7), as well as a structure we named the ELLEn module implicated in positioning of the seam-associated protein NME7 and mediating contacts between the A and B tubules of the cilia outer doublet (Fig. 8). Their positions inside the lumen of cilia outer doublets are summarized in Fig. 1. All MIP modules uncovered in our analysis are with few exceptions universally conserved in all ciliate species and excluded in non-ciliate species (Fig. 9), indicating that all these MIP architectures co-evolved with centrosomes or cilia. Hence, the MIPs likely represent early innovative structural components in the evolution of cilia with roles in the outer doublet architecture of axonemes.

**Table 1:** Summary of uncovered MT-binding modules in MIPs across *C. reinhardtii* and mammals

Domain or module	MIP	*C. Reinhardtii* paralogs	*H. Sapiens* paralogs
DM10	FAP67, RIB72	FAP67, RIB72, XP_001691061, DM10DCP	EFHC1, EFHC2, NME7, CAPS2
Mn repeat (only)	FAP363	FAP257, FAP203, FAP236	MAP6, MAP6D1, SAXO1, SAXO2, TEX45 (SAXO5), TEX26, C2ORF73, MDM1
	FAP273	-	TEX36, TEX37 (SPMIP9), C3ORF84, C1ORF100 (SPMIP3), SMRP1 (SPMIP6)
	RIB21	-	
	FAP166	-	
Mn + PYG	FAP129	-	-
NWE + Mn	FAP95	FAP68, FAP95, FAP143 FAP107	C9ORF135 (CFAP95)
	FAP107		C1ORF158 (CFAP107)
	FAP143		SPAG8
	FAP68		C11ORF1 (CFAP68)
NWE only	FAP161	FAP161	CFAP161
PYG	FAP129	FAP129, FAP252, FAP222	FAM166A (CIMIP2B), FAM166B (CIMIP2B), FAM166C (CIMIP2C), C10ORF82 (SPMIP5), SPATA48 (SPMIP7)
	FAP252		
	FAP222		
SNYG (Divergent Mn repeat + ass. helix)	FAP85	FAP90, FAP85, FAM183A, FAP144, FAP182	PIERCE1, PIERCE2, C5ORF49 (CFAP90), FAM183A (CFAP144), C20ORF85 (CIMIP1), C2ORF50, ATP6V1FNB (SPMIP1), TEX49 (SPMIP11)
	FAP182		
	FAP90		
GFG repeat	FAP77	FAP77, FAP21	EFHB, CFAP77
	FAP21		
ELLEn	FAP53	FAP53, FAP141	CFAP53
			CFAP141
	FAP141		
			TCHP

Summary of uncovered MT-binding modules in MIPs across *C. reinhardtii* and mammals. Proteins are grouped according to the two known and five novel protein modules families identified in this study: DM10 domain, Mn, PYG, and GFG repeats, and NWE, SNYG, and ELLEn modules. The identified MIP modules, except for the ELLEn module, show preference for the MT inner surface.

### Analysis of the DM10 domain family of MIPs reveals human CAPS2 as a new family member

As a starting point to study potentially shared structural modules in MIPs, sequence homologies between the known DM10 domain-containing proteins were assessed using the *C. reinhardtii* RIB72 amino acid sequence as a search query. Interestingly, besides the known DM10 domain-containing proteins, we also identified among the top significant search matches (Supplementary Fig. S1c, d) the unknown DM10 family members human calcyphosin-2 protein (CAPS2) and the *C. reinhardtii* protein of unknown function (XP_001691061) (Fig. 2a, b). The DM10 domain in CAPS2 is highly conserved (Supplementary Fig. S1a) and showed the closest homology to the DM10 domain of RIB72. This was further supported by the AlphaFold-predicted tertiary structure of the CAPS2 DM10 domain (Fig. 2c), which adopts a pleckstrin homology (PH)-like fold of seven β-strands and an α-helical region located at the C terminus, as judged by structural analysis using DALI^13^. We next asked whether CAPS2 might have functions in centrosomes or cilia. Indeed, a *CAPS2* gene co-expression analysis using the robust RNA-seq data derived from The Cancer Genome Atlas (TCGA) indicated that *CAPS2* is co-regulated markedly with ciliary genes across different multi-ciliated tissues (Fig. 2d, e). Furthermore, we found that *CAPS2* is strongly upregulated at the mRNA level in NIH3T3 fibroblasts cultured in the absence of serum to induce ciliogenesis (Fig. 2f) and at the protein level in retinal pigmented epithelial (RPE1) cell extracts as detected by immunoblotting after serum deprivation (Fig. 2g). This suggests that CAPS2 has function in processes relating to cilia biology. Indeed, immunofluorescence microscopy (IFM) using two different antibodies raised against CAPS2, revealed that endogenous CAPS2 accumulates prominently at the ciliary base in RPE1 cells and appears to be resilient to a detergent pre-extraction (Supplemental Fig. S1b), indicating that CAPS2 is firmly embedded in the centrosome compartment. Because many MIPs were shown to populate motile cilia outer doublets MTs, we further assessed the localization of CAPS2 to this subcellular compartment in human bronchial epithelial cells differentiated at air liquid interphase. Again, we found that CAPS2 was enriched at the basal bodies of motile cilia (Fig. 2h). Interestingly, CAPS2 is likely to also localize along the length of human fallopian tube cilia (Supplementary Fig. S1e), indicating that CAPS2 is universally localized to centrosomes or cilia in the human body.

### Analysis of the Mn repeat unit reveals a conserved and recurrent MT-binding motif among MIPs

Recently the *C. reinhardtii* MIP FAP363 N-terminus was found to contain arrays of short, interspersed α-helices with homology to the human Mn repeats discovered in MAP6 (also known as STOP), SAXO1, and SAXO2^6,14,15^. The Mn repeats of FAP363 and that of the *Toxoplasma gondii* SAXO1 and SAXO2 ortholog SPM1 make close contacts with the luminal microtubule lattice^1,6^. Our profile-HMM searches uncovered all the known paralogs of FAP363^4^ (Fig. 3a–c and Supplementary Fig. S2a, c). Four of the identified human proteins (MAP6, MAP6D1, SAXO1, and SAXO2) are known paralogs, whereas three additional human proteins (TEX45, TEX26, and C2ORF73) identified among the top significant search matches (Supplementary Fig. S2h), had not at the time of this analysis been assigned any structure or function. Recently, however, TEX45, now named SAXO5, was identified in the cilia outer doublets of mammalian spermatozoa^16^. Besides the elongated Mn repeat paralogs, we noticed several shorter sequences within other MIPs also showing homology, albeit less significant, to the minimal Mn repeat unit signature with syntax [TS]-[TS]-X(4)-[YF], which is predicted to form a short α-helix of approximately 7 residues. Further inspection of these homologies revealed that they represented isolated, yet conserved portions of two minimal Mn repeat units (Fig. 3a, c). All proteins bearing these doublet Mn repeat units found in *C. reinhardtii* FAP95, FAP107, FAP129, FAP143 (Supplementary Fig. S2a), and human C11ORF1, CFAP95, CFAP107, and SPAG8 (Fig. 3c, d) appeared to have co-evolved with a highly conserved N-terminal NWE motif (shown in Fig. 4) and were recently identified as MIPs^1,2,16,17^. These Mn units bind between a tubA-tubB pair in the outer doublet lattice (Fig. 3e) similarly to other Mn repeat proteins (Supplementary Fig. S2b). C11ORF1 was recently found in doublets of human airway epithelial cells and subsequently named CFAP68^17^, indicating that all identified human NWE-Mn repeat module-containing proteins are cilia proteins.

We next searched for additional Mn repeats that might exist among the plethora of MIP structures not yet accounted for by profile-HMM sequence analysis. We resorted to a Gibbs motif sampler method used successfully for recognizing and predicting short and divergent histocompatibility complex (MHC) sequence motifs^18^. These searches identified, besides the known Mn repeat-containing MIPs (Fig. 3 and Supplementary Fig. S2), six additional human proteins namely C1ORF100, SMRP1, TEX37, TEX36, C3ORF84, and MDM1 (Fig. 3c). Interestingly, four of these were recently defined as MIPs in mammalian C1ORF100 (SPMIP3), SMRP1 (SPMIP6), TEX37 (SPMIP9) and sea urchin TEX36 sperm flagella^16^ of which the experimentally determined 3D structure of TEX37 (SPMIP9) has thus far been published (Supplementary Fig. S2e). We identified multiple Mn repeats in MDM1 by both Gibbs sampling and sequence repeat analysis using the HHrepID software^19^ by automated de novo identification (Supplementary Fig. S2f), indicating that MDM1 might be an elongated type Mn repeat protein. Interestingly, MDM1 occupies the centriolar lumen of RPE1 cells^20^, supporting that MDM1 is a centriolar MIP. We also inspected the published 3D coordinates for the entire repertoire of MIPs across mammalian and *C. reinhardtii* outer doublets. This analysis revealed additional seven *C. reinhardtii* MIPs bearing a single Mn repeat unit: FAP273, FAP90, RIB21, FAP166, FAP68, FAP85, and FAP252 (Supplementary Fig. S2a–d). We have thus named these structures *inferred Mn repeat units* (Supplementary Fig. S2a, d).

### The NWE module specifies MIP association to the cilia outer doublet seam

A distinct subfamily of MIPs identified in our analysis, is represented by the five *C. reinhardtii* proteins FAP68, FAP95, FAP107, FAP143, and FAP161 and their likely mammalian orthologs CFAP95, CFAP107, SPAG8, and CFAP161. These proteins were shown to occupy the axonemal outer doublet lumen residing in close contact to the seam of the A-tubule^1^. Interestingly, using CFAP107 as forward and FAP95 as reciprocal search queries, we recovered among the top significant search matches (Supplementary Fig. S3d, e) all the seam-associated MIPs in mammals and *C. reinhardtii* (Fig. 4a, b and Supplementary Fig. S3c). These MIPs bear three highly conserved patches in tandem spanning a region comprising roughly 100 residues (Fig. 4a and Supplementary Fig. S3c) namely an N-terminal consensus sequence [ILV]-L-I-G-N-W-X-E adjoined by two C-terminal Mn repeat units (Fig. 4a, b). Hence, MIPs associated with the A-tubule seam appear to have evolved as a distinct branch with specialized structural and functional properties within the axonemal doublet luminal niche and defined by a triad of conserved co-evolved unit motifs, i.e., NWE-Mn-Mn. We have named this triad the *NWE module* after its conserved N-terminal NWE motif. Our searches also allowed us to determine the hitherto unknown closest human homologs of each of these seam-associated MIPs. We found that CFAP95, SPAG8, and CFAP161 are the human homologs of FAP95, FAP143, and FAP161 supporting the previous annotation^2^. Both C11ORF1 and CFAP107, however, are more closely related to FAP68. Hence it is conceivable that FAP107 and FAP68 are *C. reinhardtii* paralogs. All NWE module family members, despite their apparent overall structural differences, showed a similar spatial position at the seam protofilaments (A9-A10), where their two Mn repeat units are positioned between tubA and tubB in protofilament A9^1^, and the NWE motif is situated in the outermost helical region pointing into the cleft between seam protofilament A9 and A10 (Fig. 4c–g). Here the NWE motif makes contacts to residues on both A9 and A10 tubules, i.e., to three tubulin subunits (tuba-tubB pair on the A9 protofilament and an adjacent tubB on the A10 protofilament), indicating that the NWE motif is specialized towards the seam heterotypic tubulin lattice interface (Fig. 4c, f, g)). The conserved tryptophan mainly contacts the tubA subunit of A9, while the asparagine and glutamate residues engage in hydrogen bonding with the tubB subunit of the neighboring A10 protofilament. A conserved hydrophobic residue deeper buried in the tubulin lattice make contacts to an adjacent tubB subunit of A9 (Fig. 4c). In the case of C11ORF1, we found that the cilia localization was apparent from immunolabeling of human bronchial epithelial cells where C11ORF1 in motile cilia was confined to a discrete region at the outermost tip and localized at cell-cell junctions (Supplementary Fig. S2g), suggesting that C11ORF1 might function as part of the cilia distal module.

### The PYG and GFG repeats are MT inner surface-binding modules in MIPs

Another subfamily of MIPs uncovered in our analysis was discerned through the *C. reinhardtii* FAP129 query. Our remote homology searches unearthed a conserved repetitive region with short patches consisting of three highly conserved residues with the consensus sequence proline, tyrosine, and glycine (PYG) (Fig. 5a–d). These searches also yielded among the top significant matches four human proteins namely FAM166A, FAM166B, FAM166C, and C10ORF82 and three other *C. reinhardtii* MIPs XP_001689677, FAP222 and FAP252 (Fig. 5a, b and Supplementary Fig. S4k–m). FAM166A, FAM166C, and C10ORF82 were (at the time of this analysis) hitherto uncharacterized but were recently detected in the cilia outer doublets of human airway epithelial cells, *T. thermophila*, and human spermatozoa^16,17,21^, thus establishing them as MIPs. In case of human FAM166A, FAM166B, and C10ORF82, as well as *C. reinhardtii* FAP129 and XP_001689677, the PYG-containing patches are found repeated multiple times (Fig. 5a–c and Supplementary Fig. S4k). Indeed, repeat assessment of each of the PYG family member in HHrepID identified essentially all PYG repeats (Supplementary Fig. S4n–q). We have thus named this evolutionary conserved region the *PYG repeat*. Interestingly, none of the *C. reinhardtii* MIPs (FAP129, FAP222, and FAP252) adopt overall structures reminiscent of FAM166B (Fig. 5b). Rather, these PYG repeat units are found adjacent to either a kinase domain followed by two EF-hand domains in FAP252 or adjoined to four inferred Mn repeats and a C-terminal module containing two PYG motifs in tandem in FAP129 (Fig. 5b). The C-terminal PYG repeats in FAP129, however, shows greatest homology to the human C10ORF82 C-terminus, indicating that C10ORF82 might be an ortholog of FAP129. All PYG repeat units appear to adopt a loop-like fold consisting of a short α-helix adjacent to a loop containing the PYG signature motif (Fig. 5d and Supplementary Fig. S4a–h), as suggested by inspection of the coordinates of cilia outer doublets (PDBs: 6U42, 7RRO, 8IYJ). In all PYG-containing proteins, this loop points towards the microtubule lattice where it makes contacts to two adjacent tubulins via its conserved tyrosine and proline residues (Fig. 5c, d and Supplementary Fig. S4i, j). These structures were also recently confirmed experimentally in the cilia outer doublet structures of human airway epithelial cells, *T. thermophila*, and human spermatozoa^16,17,21^.

Another distinct repeat type named GFG-repeat was uncovered by our analysis of FAP21 and its human homolog EFHB. These MIPs have been shown to localize to the cilia outer doublet where they are positioned in close contact with the inner tubulin lattice between protofilament A8 and A9 adjacent to the seam^1,2^. Our remote homology searches revealed among the top significant matches (Supplementary Fig. S5e) multiple repeated sequences present in FAP21 and EFHB as well as in two additional MIP orthologs namely *C. reinhardtii* FAP77 and human CFAP77 (Fig. 6a, b and Supplementary Fig. S5a). These repeats all share a highly conserved glycine-phenylalanine-glycine (GFG) signature motif (Fig. 6a and Supplementary Fig. S5a) and each repeat comprises a short patch of approximately 31 residues that is repeated in tandem along the MT protofilament (Fig. 6c, d and Supplementary Fig. S5b). Indeed, analysis of each GFG repeat family member with HHrepID predicted essentially all GFG repeats in these proteins (Fig. 6b, d, Supplementary Fig. S5c, d). We have thus named these conserved regions GFG repeats. Each repeat spans a tubA-tubB pair where their conserved phenylalanine residues make specific contacts to tubA as shown in the case of GFG repeats in both FAP21 and EFHBB (Fig. 6e–g). In motile cilia of *Tetrahymena*, the GFG repeat protein CFAP77 was recently reported to have roles in the assembly and stability of the B-tubule at the outer junction^21^. This indicates that the GFG repeat is a structural innovation evolved within MIPs to support unique tubulin interactions.

### The SNYG module bears a divergent Mn repeat unit with MT-binding properties

As reported^2^, the C-terminus of *C. reinhardtii* MIP FAP182 that traverses the outer doublet MT lattice to contact the exterior Outer Dynein Arm Docking Complex (ODA DC), shows homology to the human MIPs PIERCE1 and PIERCE2^2^. Interestingly, closer inspection of the homologous regions across FAP182, PIERCE1, and PIERCE2, identified a short, conserved patch resembling the Mn repeat unit comprising the consensus [ST]-N-X-X-[ILVY]-G, which we have coined the SNYG motif after its conserved residues. Unlike the Mn repeat, the regions bearing this patch do not match with Mn repeat family proteins in homology searches (Supplementary Fig. S6d, e), and the consensus sequence includes a highly invariable glycine residue, suggesting that the SNYG motif represents a divergent Mn repeat unit. In addition, the SNYG motif is flanked by two short, less conserved stretches of hydrophobic amino acids as well as a predicted short SNYG-associated α-helix positioned in the C-terminal direction (Fig. 7a). We also performed remote homology searches using FAP85 as a query, which is a FAP182-related MIP component also shown to be linked to the cilia outer doublet protofilaments^1^. We found that FAP85 features an EF-hand domain in its N-terminus, but no modules have been assigned to its C-terminus. Here we identified the same minimal SNYG signature motif as in FAP182 albeit in a different subfamily of proteins comprising FAP90 among the top significant matches (Supplementary Fig. S6d, e) and to (at the time of this analysis) uncharacterized human proteins FAM183A, TEX49 (SPMIP11), ATP6V1FNB (SPMIP1), C5ORF49 (CFAP90), C20ORF85, and C2ORF50 (Fig. 7a, c). The sequence similarity between FAP182, PIERCE1, PIERCE2, and FAP85 also manifest at the tertiary structure level (Fig. 7b, d–f and Supplementary Fig. S6a, b). Inspection of the structural coordinates of the cilia outer doublets of *C. reinhardtii* FAP182 and FAP85 as well as mammalian PIERCE1 and PIERCE2 suggested that the positions of the SNYG motif at the tubA-tubB interface are also occupied by the Mn repeat units, whereas the SNYG-associated helix binds at the adjacent tubB (Fig. 7g, and Supplementary Fig. S6c). Further inspection of the SNYG module indicated that the conserved SNYG motif and SNYG-associated helix make contacts to the MT inner tubulin surface i.e., to tubA and tubB, respectively (Fig. 7g and Supplementary Fig. S6c), indicating that this module evolved specifically to bind tubulin heterodimers. Interestingly, while C5ORF49 (CFAP90), C20ORF85, C2ORF50, FAM183A, TEX49 (SPMIP11) and ATP6V1FNB (SPMIP1) had not (at the time of this analysis) been linked to cilia, the presence of a SNYG module suggests that they might have functions related to cilia biology. Indeed, recently C5ORF49, ATP6V1FNB, C20ORF85, and FAM183A were shown to occupy the lumen of cilia outer doublets in airway epithelial cells^16,17,22^. Likewise, a gene co-expression analysis using RNA-seq data obtained from the TCGA indicated that across tissues, apart from ATP6V1FNB (SPMIP1), all these gene products are co-regulated markedly with ciliary genes (Fig. 6h), further supporting their ciliary functions. Because other SNYG-bearing MIPs were shown to populate motile cilia outer doublets MTs, we assessed the localization of C2ORF50 to motile cilia in human bronchial epithelial cells by Structured Illumination Microscopy (SIM). Indeed, C2ORF50 was found to be enriched along the length of motile cilia and its antibody specificity confirmed by CRISPR-Cas9 KO (Fig. 7i, Supplementary Fig. S6g). These same SNYG module-containing proteins also show strong enrichment in human fallopian tube motile cilia (Supplementary Fig. S6f).

### ELLEn is a conserved NME7- and tubulin-binding module found in CFAP53, CFAP141, and TCHP

*C. reinhardtii* FAP53 belongs to a group of axonemal outer junction-spanning MIPs comprising FAP53, FAP127, and FAP141 that are believed to serve architectural roles in specifying the position of the B-tubule adjacent to the seam of the A-tubule^1^. Interestingly, they also interact with the above-mentioned NWE module-containing MIPs (FAP68, FAP95, and FAP143) likely to enable fastening to the seam. Our remote homology searches recovered homologous regions in the FAP53 and FAP141 proteins of a region spanning approximately 70 residues (Supplementary Fig. S8a). Our searches also uncovered among the top significant matches (Supplementary Fig. S7d) homologous regions in the human homologs, CFAP53 and CFAP141, and the centrosomal protein trichoplein (TCHP) not previously identified as a MIP (Fig. 8a, b), suggesting that these proteins are not only functionally but also evolutionary and structurally related. We have named this module ELLEn to emphasize the presence of conserved signature leucine (L) and glutamate (E) residues within the N-terminus of FAP53 and CFAP53 (Fig. 8a and Supplementary Fig. S8a). The ELLEn module shows strict homology both in terms of residues and peptide steric distance of the core residues (Supplementary Fig. S8a) i.e., R, L, E/D, Φ (Phi), E, Φ (Phi), E, and Φ (Phi), as evidenced by the syntax: [LIV]-X(3,4)-[RK]-X(23,29)-[W]-X(24,31)-[LIV]-X(2)-R-X(3)-[LIV]-X(2)-[LIVMFY]-[LIVMFY]-X(2)-E-X(3)-[LIVFMY]-X(2)-E. The syntax covers approximately 70 residues (CFAP53 residue 5-80) and matches only with CFAP53, TCHP, and CFAP141 in humans. We noticed that among the homologous filamentous MIPs (fMIPs), *C. reinhardtii* FAP53 and FAP141, but not FAP127, bind to FAP67 at the seam. Likewise, in humans the orthologs CFAP53 and CFAP141, but not MNS1 (FAP127 in *C. reinhardtii*), bind to the FAP67 homolog of NME7. We therefore inspected the regions in CFAP53 and CFAP141 responsible for their binding to NME7. In both cases we found that the ELLEn module in CFAP53 and CFAP141 encompasses the regions binding NME7 (Fig. 8c). Interestingly, further analysis of the FAP53 and FAP141 ELLEn module positions in the coordinates of the *C. reinhardtii* outer doublet showed that the conserved hydrophobic residues in the outermost N-terminus within the ELLEn module are responsible for tubulin contacts on both the A and B tubules (Fig. 8c), raising the possibility that the ELLEn modules of FAP53 and FAP141 (and the mammalian CFAP53 and CFAP141) play critical roles in the assembly of the cilia outer doublet. Supporting this view, we found that human CFAP53 localizes along the axoneme in motile cilia while NME7 was more enriched at the cilia tip (Supplemental Fig. S9a, b).

NME7 and CFAP53 were recently found mutated in patients with *situs inversus totalis*^9,23,24^. In the case of NME7, patients have an exon 10 in-frame deletion in NME7, which corresponds to several amino acids residing in the NME7-CFAP53 and NME7-CFPA141 interfaces with several of their contact sites affected (Supplemental Fig. 8g.). Because the ELLEn module in TCHP shows high homology to the other family members and the same overall helical secondary structure, we asked whether the N-terminal portion of TCHP containing the ELLEn module might also reside in a protein complex with full length NME7. Indeed, using the AlphaFold-Multimer suite^25^, we found that the TCHP ELLEn module was predicted to bind FAP67 and NME7 similar to the ELLEn module in FAP53, CFAP53, FAP141, and CFAP141 (Fig. 8d and Supplementary Fig. S8g). This indicates that the ELLEn module is a universal NME7-binding module. The ELLEn module in TCHP shows similar contacts to NME7 and FAP67 as for the CFAP53-NME7 and CFAP141-NME7 complexes (Fig. 8e and Supplementary Fig. S8f, g).

We co-discovered TCHP as a centrosomal component^26,27^ by protein correlation profiling and in several large scale interactome and two-hybrid studies, TCHP is found as a potential NME7 binding partner^28–32^ (Supplementary Fig. S8b), hinting that TCHP associate with centrosomes, centrioles, or centriolar satellites in complex with NME7 possibly through interaction with the ELLEn module. To test the predictive power of our analysis together with the previous published data, we assessed the possible NME7-TCHP interaction by FLAG pulldown analysis using FLAG-NME7 fragments expressed in HEK293T cells. Indeed, we found that TCHP co-eluted readily with FLAG-NME7 dependent on the presence of its C-terminal NDPk7B region (Fig. 8f, g). Likewise, full length but not an ELLEn-deleted version of FLAG-TCHP could reciprocally pull down NME7 (Fig. 8h, i), thus establishing that NME7 and TCHP physically interact through the ELLEn module of TCHP. Localization of TCHP at centrosomes and CFAP53 at nodal basal bodies indicate a role in centriole assembly or function. To test this, we profiled proteins by proteomics from centrosomes isolated from cells induced to undergo PLK4-mediated centriole biogenesis. An increased level of PLK4 was achieved by inhibiting E3 ubiquitin ligase mediated degradation of PLK4 using the NEDD8-activating enzyme inhibitor MLN4924. Profile data obtained for centrosome-associated proteins confirmed an increase in PLK4 together with SAS6, STIL, and additional early centriole biogenesis factors (Fig. 8j). NME7 and TCHP were recruited with comparable dynamics in support of the notion that the two proteins take part in centriole biogenesis (Fig. 8j). Co-localization of NME7 and TCHP at the centrosome was further supported by immunofluorescence microscopy (Supplemental Fig. S8c). To further investigate the biological significance of the emerging interplay between NME7 and TCHP, we depleted NME7 from RPE1 cells using CRISPR targeted against three exons within *NME7*. All three gRNAs could efficiently knockout NME7 (Supplementary Fig. S7a–c) and deplete NME7 from the ciliary base (Supplementary Fig. S7i). Interestingly, loss of NME7 also appeared to co-deplete TCHP (Supplementary Fig. S7a–c), suggesting that the stability of TCHP is depending on NME7. Because TCHP loss has been associated with gross chromosome segregation errors and ensuing aneuploidy as well as premature cilia formation in cycling cells^33,34^, we examined whether TCHP co-depletion after NME7 loss would recapitulate such phenotypes. Indeed, we found that NME7-depleted cycling cells showed mild cell cycle accumulation in G1 phase (Supplementary Fig. S7g, h), which was accompanied by increased cilia formation (Supplementary Fig. S7e, f). The NME7-depleted cells also showed increased levels of chromosome segregation errors and micronuclei (Supplementary Fig. S7j–l), supporting that TCHP co-depletion in NME7 deficient cells phenocopies that of TCHP-deficient cells^33,34^.

## Discussion

Here we identified hitherto unknown MT-binding modules, which alone or in combination with known MT-binding modules were not recognized in MIPs. Together, they represent seven evolutionary conserved protein families in eukaryotes. These are the DM10 domain, the Mn, PYG, and GFG repeats, and the NWE, SNYG, and ELLEn modules. We found that CAPS2 contains a hitherto unknown DM10 module (Fig. 2) and our subsequent validation of CAPS2 suggested that it is a basal body component of both primary and motile cilia (Figs. 1, 2 and Supplementary Fig. S1). We further uncovered remote homologies to 11 additional MIPs in *C. reinhardtii* and to 32 MIPs in humans (summarized in Table 1, Fig. 1, and Supplementary Table S1). Interestingly, the most prevalently conserved unit is the Mn repeat, which appears to have been repurposed across MIPs, either as a repeated MT-binding unit by itself or in combination with another N-terminal positioned MT-binding motif, as shown here for protein families with the NWE or SNYG modules or the PYG repeats. The Mn repeat is found thus far in MAP6 associated with the lumen of cytoplasmic MT^3,35,36^ and in multiple MIPs associated with motile cilia outer doublet MT in sperm tail, trachea, and bronchia from various organisms^2,16,17,21,22,37^, suggesting that it likely also exists in MIPs associated with other types of MT structures.

Our unearthing of seam-binding modules, either binding directly (NWE module) or indirectly (ELLEn module) (Figs. 4 and 8), strengthen the notion that the MT seam likely marks and transduces the starting site for the construction of cilia MT doublet and possibly also the centriole triplet structures. NWE modules bind directly to the MT seam (Fig. 4), which could help to stabilize doublet microtubules and enable the seam to transmit its unique position in the microtubule lattice via the seam-associated MIPs. It is conceivable that the NWE module together with the two adjoining Mn repeat units help to stabilize the positioning of the NWE-containing helix inside the seam cleft. One exception to this notion is FAP161 that bears the NWE motif but lack the Mn repeats, indicating that the NWE motif is sufficient to bind to the seam. FAP161 was suggested to occupy the A-tubule of centriole triplets and predicted to occupy the seam position in the ciliary outer doublet seam^12^. This indicates that the NWE module is evolutionarily conserved in both axoneme and centriole structures. Other NWE-containing MIPs are found in complex with NME7, which is universally associated with the A-tubule seam in axonemes and centrioles across eukaryotes^38,39^. FAP67 and NME7 form complexes with the NWE-containing proteins FAP68, FAP95, CFAP95, FAP143, and SPAG8, as well as with the fMIPs, FAP53, CFAP53, FAP141, and CFAP141. Because they link the A- and B-tubule via the outer junction intersection, these fMIPs are thought to be crucial for the overall outer doublet assembly^1^ (Fig. 8c). The shared ELLEn module among the fMIPs (Fig. 8), which facilitates their mutual contacts with FAP67 and NME7 could explain the unique position of FAP67 and NME7 at the seam possibly facilitated by the NWE proteins. Interestingly, the ELLEn module was also found in TCHP, possibly explaining its localization at the centrosome and binding to NME7 (Fig. 8). The shared MIP features of TCHP and CFAP53 and their mutual binding to NME7, also provide possible mechanisms for future studies on how TCHP could modulate ciliogenesis by interfering with the CFAP53 tripartite lateral interaction between the A10, A11 and B1 protofilaments at the outer junction (Figs. 4g and 8c). Another MT-binding family uncovered, the PYG repeats, was predicted in three *C. reinhardtii* and four human proteins including the MIP FAM166B, thus identifying FAP222, FAP252, or FAP129 as the likely *C. reinhardtii* orthologs of human FAM166A-C and C10ORF82 (Fig. 5). An N-terminal module we dubbed the SNYG module, is a divergent Mn repeat unit attached to an α-helix extension, which was detected in five *C. reinhardtii* and seven human proteins (Fig. 7). Collectively, our identification of the MT-binding modules in several MIPs support the notion that MIPs bear specialized MT-binding structures, which help to explain how MIPs are specifically targeted and bound to the inner surface of the MT lumen. Interestingly, the identified MIP modules appear to have co-evolved with centrosomes and cilia (Fig. 9), indicating that these modules evolved to support MT to function specifically in centrosomes and cilia.

## Methods

### Computational homologous sequence searching and sequence analysis

MIP full-length FASTA sequences were retrieved from the Uniprot database. Inspection of their primary structures (Supplementary Table S1) suggested that the great majority had not (by the time of this survey) been assigned evolutionary or structural relationships to other proteins. This is likely due to the intrinsically disordered structure (IDP) and coiled-coil propensity of many MIPs (Supplementary Table S1) as suggested by analysis using the IUpred2a, DISOPRED, and Coils algorithms^40–42^. To mitigate spurious results and statistical bias, only regions devoid of coiled-coils and masked for compositional complexity or IDP were included in the searches. The resulting sequences were utilized to construct Multiple Sequence Alignments (MSAs) through multiple reiterative searches using the HHblits search approach^43^ against the UniRef30 database with 5 iterative searches as default. An E-value cutoff of E = 0.01 was applied for MSA generation. Constructed MSA’s were used to build HMMs suitable for iterative profile-to-profile searches (profile-HMM) with the open-source software package HH-suite (https://github.com/soedinglab/hh-suite) and HHpred^44^ to retrieve remote homologous sequences in the protein PFAM database of hidden Markov models. Both local and global realignment search modes were used. Examples of the profile-HMM search results are deposited at the https://github.com/Schoulab/MIP_structures/tree/main/HHsearch repository. To validate remote homologies identified by these initial (forward) searches, matched sequences (E < 0.01) were extracted and used as new search queries (reciprocal searches) in another round of MSA generations and HMM-to-HMM searches against the PFAM database. Only matches that for reciprocal searches recapitulated the initial search query were considered reliable. To further validate remote homology matches, reciprocal 3D models of candidate homologies were assessed with the AlphaFold method using the ColabFold software package available at https://github.com/sokrypton/ColabFold^45^. The MMseqs2 search engine^46^ was used to build the MSAs used for profile-HMM searches and subsequent evo blocks. No PDB templates were employed. Calculated Viterbi raw score values for the score distribution plots was for each query obtained from HHsearch outputs along with the E-values. Finally, to further validate the robustness of the MIP profile-HMM searches, we reassessed searches by manually inspecting MSAs for corrupted regions using the filter options for HHblits query MSAs provided by the HH-suite package. Briefly, the HHblits query MSA was reduced to include a set of the most diverse >30 sequences across species using the hhfilter (-diff) option. As sequences that contain non-homologous stretches tend to be the most dissimilar to the query sequence, these are usually retained upon using this filter option. Next the resulting (filtered) MSA was trimmed for inserts and non-homologous extensions using the remove all insert option (-r) to produce a master-slave alignment suitable for identification for non-homologous regions. After visual inspection of the resulting (filtered, trimmed) MSA, problematic (non-homologous or short) regions were removed from the alignment and subsequently the resulting (filtered, trimmed, and curated) MSA was used to build a new HMM for another round of profile-HMM searches. In all cases, the initially identified MIP structure families were recovered using the curated MSAs. The scripts for the filtering procedure of MIP MSAs are deposited at the https://github.com/Schoulab/MIP_structures/tree/main/Scripts repository. Multiple sequence alignments were built by MAFFT^47^ followed by careful manual adjustments based on profile-profile alignment, secondary structure information, and structural alignment. The consensus of the alignment was calculated and colored by the ClustalW^48^. Consensus secondary structures were predicted using the JPred program^49^.

### Gibbs sampling analysis

The short Mn repeat unit sequence is too short and divergent to yield significant matches using profile-HMM analysis. We therefore resorted to a Gibbs motif sampler method used successfully for recognizing and predicting weak histocompatibility complex (MHC) sequence motifs^18^. We used the expanded Mn motif alignment (Supplementary Fig. S2c) as a training set for the Mn repeat unit weight-matrices applied to identify potential Mn repeat-containing candidates. Each search was performed using the motif length parameter of 12 and with the Henikoff & Henikoff clustering method as matrix parameters. Only matches with a prediction score >19 were used.

### 3D structural assessment

Coordinates of the entire cilia outer doublets from all studies providing Protein Data Bank structure (PDB) results (PDB: 6U42, 7RRO, 8G2Z, 8G3D, 8SNB, 7UNG, 8IYJ, 8J07, and 7MIZ) were retrieved from the PDB. Structures were analyzed in BIOVA Discovery Studio Visualizer (19) or PyMol (20).

### Phylogenetic analysis

We assessed each MIP family alignment for maximum likelihood (ML) phylogenetic analyses using the maximum-likelihood method IQ-Tree v.2.0^50^ to estimate phylogenetic trees. In our analyses, we utilized molecular evolution models determined through ModelFinder^51^ integrated into IQ-Tree. This approach identified an optimal partitioning scheme and the best model for each partition. To evaluate the support for the resulting topology, we conducted 1,000 ultrafast bootstrap replicates^52^.

### Antibodies

For immunoblot analysis, the following primary antibodies were used (dilutions in parenthesis): rabbit anti-TCHP (1:500) (25931-1-AP, ProteinTech), rabbit anti-NME7 (1:500) (NBP2-42888, Novus Biotechnology), rabbit anti-FLAG (1:1000) from Invitrogen, rabbit anti-CAPS2 (HPA040004) (1:500) from Sigma or (11924-1-AP) (1:500) from ProteinTech, and rabbit anti-GAPDH (1:2,000) from CellSignal. Secondary antibodies used for immunoblotting: horseradish peroxidase-conjugated goat anti-mouse (P0447, 1:4,000) or swine anti-rabbit (P0399, 1:4,000) from Dako. For IFM analysis, the following primary antibodies were used: mouse anti-ARL13B (sc-515784, Santa Cruz), mouse anti-γ-tubulin (1:2000) (MABT163, Sigma), from rabbit anti-TCHP (1:500) (25931-1-AP, ProteinTech), rabbit anti-NME7 (1:500) (NBP2-42888, Novus Biotechnology), mouse anti-FLAG M2 (1:1000) from Sigma, rabbit anti-CAPS2 (HPA040004) (1:500) from Sigma or (11924-1-AP) (1:500) from ProteinTech, mouse anti-Ac-tubulin (T7451, Sigma), mouse anti-CNTRL (sc-365521, Santa Cruz), rabbit anti-C2orf50 (HPA067681, Atlas Antibodies), rabbit anti-C11orf1 (HPA038410, Sigma Aldrich). Secondary antibodies used for IFM (all from Invitrogen and diluted 1:600, catalog number in parenthesis): Alexa Fluor 350-conjugated donkey anti-mouse (A-10035) or donkey anti-rabbit (A-10039); Alexa Fluor 488-conjugated donkey anti-mouse (A-21202), donkey anti-rabbit (A-21206) or donkey anti-goat (A-11055), Alexa Fluor 568-conjugated donkey anti-mouse (A-10037), donkey anti-rabbit (A-10042) or donkey anti-goat (A-11057).

### PCR, cloning procedures, and plasmids

Plasmids encoding full length or truncated versions of FLAG-TCHP were generated by PCR with relevant primers and human TCHP plasmid as template (Origene) followed by cloning into pFLAG-CMV2 (Clontech) by standard procedures. Primers used for cloning human TCHP were FLAG-TCHP full length FW: AAAAA GCGGCCGCAATGGCGCTCCCGACGCTGC, RV: AAAAAGATATCTCAGTTCCAAGCAATTTTTG. FLAG-TCHP ELLEn-deleted FW: AAAAAGCGGCCGCATTGCAGGAAAGAAGAATC and RV: AAAAAGATATC TCAGTTCCAAGCAATTTTTG. Plasmids encoding FLAG-tagged full-length or truncated human NME7 was a kind gift from Robert Z. Qi. For CRISPR-Cas9 mediated knockout of NME7, 20 nt oligos targeting exon 1, 6, and 8 in *NME7* (5′ - GATTCGTTTTCATTGCAGAG-3′, 5′-GAGTTGTTTTTTCCTTCAAG-3′, and 5′ TATGGATCGGGTTAATGTT G-3′) were cloned into pSpCas9(BB)-2A-GFP (px458) using a published procedure^53^. *Escherichia coli* TOP10 was used for transformation and plasmid amplification, and plasmids were purified using NucleoBond Xtra Midi EF Kit from Macherey-Nagel. sgRNA targeting the C2ORF50 locus was designed in silico using CRISPOR tool to assess specificity, predicted efficiency, and predicted off-targets^54^. For CRISPR-Cas9 mediated knockout of C2ORF50, a 20 nt oligo targeting C2ORF50 (5′ - GGUACCGAUUGCCCCCCACC -3′ was used. A scrambled oligo 5′-GCACUACCAGAGCUAACUCA-3′ was used as control.

### RNP complex generation

Synthetic modified sgRNA (2′-O-methyl analogs and 3′ phosphorothioate internucleotide linkages in first three nucleotide at both 5′ and 3′ ends) (Synthego) for the C2ORF50 locus and scrambled control were resuspended in nuclease-free 1X TE buffer. Ribonucleoprotein (RNP) complexes were made by 10 min RT incubation 1:1 with TrueCut Cas9 Protein v2 (Invitrogen) diluted in Buffer R (Invitrogen).

### gDNA Extraction and ICE Validation

500,000 cells from the knockout pool, and the scrambled control were pelleted (300 × *g*, 5 min) and gDNA extracted using GeneJET Genomic DNA Purification Kit (Thermo Scientific). The *C2ORF50* locus was amplified by PCR from the knockout and scrambled gDNA using Platinum SuperFi II PCR Master Mix (Invitrogen). PCR products were purified (QIAquick PCR Purification Kit - QIAGEN) and Sanger sequenced. Knockout efficiency was determined by Inference of CRISPR Edits (ICE) analysis (Synthego). The result showed 90% indel, model fit of 93, and a knockout score of 82.

### Cell culture and transfections

HEK293T cells were cultured at 37 °C in Dulbecco’s modified Eagle’s medium (DMEM, Gibco), supplemented with 10% heat-inactivated fetal bovine serum (FBS, Gibco) and penicillin-streptomycin (Gibco), under conditions of 5% CO2 and 95% humidity. RPE1 cells, derived from the immortalized hTERT RPE1 cell line (ATCC CRL-4000), were cultured in a mixture of 45% DMEM and 45% F-12 (Ham; Sigma), supplemented with 10% FBS and penicillin-streptomycin. Culture passages were performed every 3–4 days. For the transfection of hTERT-RPE-1 cells with plasmids for immunofluorescence microscopy (IFM), cells were cultivated in six-well plates until reaching approximately 90% confluence. Transfection was carried out using 1 μg of plasmid, employing FuGene 6 (E2692, Promega) as the transfection reagent. The cells were then incubated for 24 h, followed by an additional 24 h of serum deprivation. In the case of HEK293T cells, plasmid transfection involved 8 μg of DNA in a 10 cm dish, with FuGene 6 as the transfection reagent. The cells were incubated for an additional 24 h.

### Human bronchial airway cell culture & electroporation

hTERT immortalized human bronchial epithelial cells (BCi-NS1.1) were cultured in PneumaCult-Ex Basal Medium (STEMCELL Technologies) as previously done^55^. At 80% confluency, cells were detached by addition of Trypsin-EDTA (0.05%) (Gibco). RNP complex was transfected into cell suspension using NEON transfection system (Invitrogen) at a concentration of 6 pmol RNP complex per 100,000 cells. Transfected cells (including scrambled control) were cultured in PneumaCult-Ex Basal Medium (STEMCELL Technologies).

At confluency, transfected cells were passaged and seeded onto HTS Transwell 96 Well Permeable Support System with 0.4 μm pore size, polyester membrane (Corning) at 25,000 cells per well. Cells were grown until confluent (3–4 days) in PneumaCult-Ex Basal Medium (STEMCELL Technologies) before removal of apical media (Air-lift) and culture in PneumaCult-Air Liquid Interphase Medium (STEMCELL Technologies). Cells were differentiated during 28 days with media change in the Transwell basal chamber every 2 days.

### Immunofluorescence microscopy

The IFM analysis procedure involved the following steps: cells cultured on glass coverslips were rinsed in ice-cold PBS, then treated with a 4% paraformaldehyde (PFA) solution. Subsequently, permeabilization was carried out using a permeabilization buffer (PBS containing 0.1% (v/v) Triton-X100 and 1% (w/v) bovine serum albumin), and the cells were subjected to IFM following the specified protocol^55,56^ For IFM analysis of FLAG-TCHP, FLAG-NME7 as well as endogenous TCHP and NME7, cells were treated the same way except that they were subjected to a brief pre-extraction with gentle incubation with ice cold CSK pre-extraction buffer (10 mM Hepes, pH 7.0, 100 mM NaCl, 300 mM sucrose and10 mM EDTA) containing 0.5% Triton X-100) for 5 min on ice prior to fixation with ice cold 4% PFA. Following the fixation step imaging was performed using a Zeiss Observer Z1 microscope, and the acquired images underwent processing for publication using Adobe Photoshop CS4 version 11.0.

### Human Airway Bronchial Immunofluorescence staining and antibodies

Human bronchial BCi-NS1.1 cells differentiated at Air Liquid Interphase were directly fixed on Transwell filters with methanol (Fisher Chemical, Methanol Histological Grade) kept at −20 °C for 30 min^56^. The filters were then washed three times for 7 min, blocked with 4% BSA and 0.05% tween in PBS for 1 h at room temperature (RT) and incubated with primary antibodies diluted in blocking buffer overnight at 4 °C. The following primary antibodies were used: mouse IgG2b anti-Ac-tubulin (1:150, Sigma Aldrich, T7451), mouse IgG1 anti-CNTRL (1:100, Santa Cruz, sc-365521), rabbit IgG anti-C2orf50 (1:50, Atlas Antibodies, HPA067681), rabbit IgG anti-C11orf1 (1:50, Sigma Aldrich, HPA038410), and rabbit IgG anti-CAPS2 (1:50, Proteintech, 11924-1-AP). After an overnight incubation, the filters were washed three times for 7 min at RT. For secondary antibodies, goat Alexa 488 anti-mouse IgG2b (1:200, Thermo, A21141) was used to label anti-Ac-tubulin, alpaca VHH fragment Cy3^TM^ anti-rabbit (1:200, Jackson ImmunoResearch, 611-164-215) was used to label anti-C2orf50, anti-C11orf1, and anti-CAPS2, and goat Alexa 647 anti-mouse IgG1 (1:200, Thermo, A21240) was used to label anti-CNTRL. Secondary antibodies were incubated for 1 h at RT, washed three times for 7 min and mounted in Prolong gold mounting medium (Invitrogen). All washing steps were performed with 0.05% tween in 1x PBS.

### 3D-SIM micrograph acquisition

3D-SIM micrographs were collected using a ZEISS Elyra 7 system (Carl Zeiss Microscopy) equipped with alpha Plan-Apochromat 63x/1.40 Oil Koor M27 Var2 objective and Duolink 4.2 CL HS pco.edge sCMOS cameras. The fluorophores were excited with lasers wavelengths 488 (500 mW power, range 13–15%, exposure times 10–15 ms); 561 (500 mW power, range 25–70%, exposure times x-y msec), 647 (500 mW, range 14–18%, exposure times 25–40 msec). The emission wavelengths were collected by passing through band-pass filter 495–550, long-pass 655 nm. For each individual z slice, thirteen raw images were acquired to collect phase information for Lattice SIM reconstruction. Raw data were reconstructed using the SIM module of ZEN Black 3.0 software version 16.0.18.306 with the auto sharpness filter function. Channel alignment was conducted using a .bin calibration file generated from data collected using Tetraspec beads calibration slide (Carl Zeiss Microscopy).

### Flow cytometry

Cell cycle profiles, as determined by fluorescent staining with propidium iodide (PI), were monitored by flow cytometric analysis using the BD FACSMelody Cell Sorter. Cycling wild type or NME7-depleted cells were harvested, fixed in 70% ethanol, and stained with PI supplemented with RNase A (10 μg/ml) for 30 min at 37 °C. The flow cytometry results were analysed using FlowJo™ v10.8 Software (BD Life Sciences).

### Immunoprecipitation and immunoblotting

HEK293T cells were transfected the day before immunoprecipitation (IP). Cells were harvested in ice cold EBC buffer (140 mM NaCl, 10 mM Tris-HCl, 0.5% NP-40 and protease inhibitor cocktail (Roche)). The subsequent IP was performed with 10 µl Anti-FLAG (M2) conjugated magnetic beads for 1 h, and Immunocomplexes were washed five times in EBC buffer before elution with 1x FLAG peptide (Sigma). Eluted FLAG-protein complexes were purified further by micropore filter centrifugation. Analysis by SDS-PAGE and immunoblotting with relevant antibodies was performed using the Novex system from Invitrogen and by following the protocol supplied by the vendor. Blots were incubated in primary antibodies at appropriate dilutions, incubated with relevant horse radish peroxidase conjugated secondary antibodies. Images were processed in Adobe Photoshop CS6. Immunoblot source data are provided with this paper.

### Quantitative analysis of centrosome-associated proteins during centriole biogenesis

Human KE37 lymphoblast cells were grown in SILAC RPMI 1640 medium (Thermo Scientific) with normal L-Lysine (Lys0) and L-arginine (Arg0), or isotope-labeled L-Lysine ^2^H_4_ (Lys4) and L-arginine ^13^C_6_^14^N_4_ (Arg6), or ^13^C_6_^15^N_2_ (Lys8) and ^13^C_6_^15^N_4_ (Arg10) (Sigma-Isotope, St Louis, MO) for at least one week to obtain complete isotope incorporation. To induce centriole biogenesis, the level of PLK4 was increased by preventing E3 ubiquitin ligase-mediated PLK4 degradation in cells using the NEDD8-activating enzyme inhibitor MLN4924 at a final concentration at 1 µM for 6 or 18 h. Centrosome-associated proteins were then identified and quantified from untreated or inhibited SILAC-labeled cells by mass-spectrometry-based proteomics analysis of isolated centrosomes as previously described^27^. The experiments were performed in two replicates.

### Reporting summary

Further information on research design is available in the Nature Portfolio Reporting Summary linked to this article.

### Supplementary information

Supplementary Information

Reporting Summary

### Source data

Source Data

## Data Availability

The mass spectrometry proteomics data have been deposited to the ProteomeXchange Consortium via the PRIDE partner repository with the dataset identifier PXD037582. All data used but not produced in this study are available in the PDB database: 8G2Z (48-nm *Tetrahymena thermophila* WT doublet), 6U0H (48-nm *Tetrahymena* WT doublet), 6U42 (48-nm *Chlamydomonas reinhardtii* WT doublet), 8GLV (48-nm *Chlamydomonas reinhardtii* WT doublet), 8IYJ (48-nm mouse WT doublet), 8SNB (48-nm sea urchin WT doublet), 7UNG (48-nm human WT doublet), 8J07 (96-nm human WT doublet and associated axonemal complexes), and 7RRO (48-nm Bovine WT doublet). Datasets for gene co-expression analysis are available http://gepia2.cancer-pku.cn/#dataset, The Cancer Genome Atlas (TCGA https://www.cancer.gov/ccg/research/genome-sequencing/tcga), and the Genotype-Tissue Expression (GTEx https://gtexportal.org/home/).

## Code availability

The Bash code for profile-HMM searches is available on Github https://github.com/soedinglab/hh-suite and 10.5281/zenodo.10683945. MIP profile-HMM searches raw output files have been deposited as HHR files on Github https://github.com/Schoulab/MIP_structures/tree/main/HHsearch. In addition, we have deposited the curated MIP query alignment scripts (including the alignments) on a Github repository https://github.com/Schoulab/MIP_structures/tree/main/Scripts. Source data are provided with this paper.

## References

[1] Ma M (2019). Structure of the decorated ciliary doublet microtubule. Cell.

[2] Gui, M. et al. De novo identification of mammalian ciliary motility proteins using cryo-EM. *Cell* **184**, 5791–5806.e19 (2021). 10.1016/j.cell.2021.10.007 PMC8595878 34715025

[3] Wang X (2021). Cryo-EM structure of cortical microtubules from human parasite Toxoplasma gondii identifies their microtubule inner proteins. Nat. Commun..

[4] Gui M, Wang X, Dutcher SK, Brown A, Zhang R (2022). Ciliary central apparatus structure reveals mechanisms of microtubule patterning. Nat. Struct. Mol. Biol..

[5] Leung MR (2021). The multi-scale architecture of mammalian sperm flagella and implications for ciliary motility. EMBO J..

[6] Imhof S (2019). Cryo electron tomography with volta phase plate reveals novel structural foundations of the 96-nm axonemal repeat in the pathogen Trypanosoma brucei. Elife.

[7] Khalifa AAZ (2020). The inner junction complex of the cilia is an interaction hub that involves tubulin post-translational modifications. Elife.

[8] Ichikawa M (2017). Subnanometre-resolution structure of the doublet microtubule reveals new classes of microtubule-associated proteins. Nat. Commun..

[9] Narasimhan V (2015). Mutations in CCDC11, which encodes a coiled-coil containing ciliary protein, causes situs inversus due to dysmotility of monocilia in the left-right organizer. Hum. Mutat..

[10] Ichikawa M (2019). Tubulin lattice in cilia is in a stressed form regulated by microtubule inner proteins. Proc. Natl. Acad. Sci. USA.

[11] Dymek EE (2019). PACRG and FAP20 form the inner junction of axonemal doublet microtubules and regulate ciliary motility. Mol. Biol. Cell.

[12] LeGuennec M, Klena N, Aeschlimann G, Hamel V, Guichard P (2021). Overview of the centriole architecture. Curr. Opin. Struct. Biol..

[13] Holm L (2022). Dali server: structural unification of protein families. Nucleic Acids Res..

[14] Dacheux D (2015). Human FAM154A (SAXO1) is a microtubule-stabilizing protein specific to cilia and related structures. J. Cell Sci..

[15] Bosc C, Oenarier E, Andrieux A, Job D (1999). STOP proteins. Cell Struct. Funct..

[16] Leung MR (2023). Structural specializations of the sperm tail. Cell.

[17] Gui M (2022). SPACA9 is a lumenal protein of human ciliary singlet and doublet microtubules. Proc. Natl. Acad. Sci. USA.

[18] Nielsen M (2004). Improved prediction of MHC class I and class II epitopes using a novel Gibbs sampling approach. Bioinformatics.

[19] Biegert A, Soding J (2008). De novo identification of highly diverged protein repeats by probabilistic consistency. Bioinformatics.

[20] Van de Mark D, Kong D, Loncarek J, Stearns T (2015). MDM1 is a microtubule-binding protein that negatively regulates centriole duplication. Mol. Biol. Cell.

[21] Kubo S (2023). Native doublet microtubules from Tetrahymena thermophila reveal the importance of outer junction proteins. Nat. Commun..

[22] Zhou L (2023). Structures of sperm flagellar doublet microtubules expand the genetic spectrum of male infertility. Cell.

[23] Reish O (2016). A homozygous Nme7 mutation is associated with situs inversus totalis. Hum. Mutat..

[24] Silva E (2016). Ccdc11 is a novel centriolar satellite protein essential for ciliogenesis and establishment of left-right asymmetry. Mol. Biol. Cell.

[25] Evans, R. et al. Protein complex prediction with AlphaFold-Multimer. *bioRxiv*, 2021.10.04.463034 (2021).

[26] Ibi M (2011). Trichoplein controls microtubule anchoring at the centrosome by binding to Odf2 and ninein. J. Cell Sci..

[27] Jakobsen L (2011). Novel asymmetrically localizing components of human centrosomes identified by complementary proteomics methods. EMBO J..

[28] Boldt K (2016). An organelle-specific protein landscape identifies novel diseases and molecular mechanisms. Nat. Commun..

[29] Huttlin EL (2015). The BioPlex network: a systematic exploration of the human interactome. Cell.

[30] Luck K (2020). A reference map of the human binary protein interactome. Nature.

[31] Rolland T (2014). A proteome-scale map of the human interactome network. Cell.

[32] Rual JF (2005). Towards a proteome-scale map of the human protein-protein interaction network. Nature.

[33] Lauriola A (2020). Depletion of trichoplein (TpMs) causes chromosome mis-segregation, DNA damage and chromosome instability in cancer cells. Cancers (Basel).

[34] Inoko A (2012). Trichoplein and Aurora A block aberrant primary cilia assembly in proliferating cells. J. Cell Biol..

[35] Cuveillier C (2020). MAP6 is an intraluminal protein that induces neuronal microtubules to coil. Sci. Adv..

[36] Foster HE, Ventura Santos C, Carter AP (2022). A cryo-ET survey of microtubules and intracellular compartments in mammalian axons. J. Cell Biol..

[37] Walton T (2023). Axonemal structures reveal mechanoregulatory and disease mechanisms. Nature.

[38] Li S, Fernandez JJ, Marshall WF, Agard DA (2012). Three-dimensional structure of basal body triplet revealed by electron cryo-tomography. EMBO J..

[39] Greenan GA, Keszthelyi B, Vale RD, Agard DA (2018). Insights into centriole geometry revealed by cryotomography of doublet and triplet centrioles. Elife.

[40] Jones DT, Cozzetto D (2015). DISOPRED3: precise disordered region predictions with annotated protein-binding activity. Bioinformatics.

[41] Meszaros B, Erdos G, Dosztanyi Z (2018). IUPred2A: context-dependent prediction of protein disorder as a function of redox state and protein binding. Nucleic Acids Res..

[42] Lupas A, Van Dyke M, Stock J (1991). Predicting coiled coils from protein sequences. Science.

[43] Remmert M, Biegert A, Hauser A, Soding J (2011). HHblits: lightning-fast iterative protein sequence searching by HMM-HMM alignment. Nat. Methods.

[44] Meier A, Soding J (2015). Automatic prediction of protein 3D structures by probabilistic multi-template homology modeling. PLoS Comput. Biol..

[45] Mirdita M (2022). ColabFold: making protein folding accessible to all. Nat. Methods.

[46] Steinegger M, Soding J (2017). MMseqs2 enables sensitive protein sequence searching for the analysis of massive data sets. Nat. Biotechnol..

[47] Katoh K, Misawa K, Kuma K, Miyata T (2002). MAFFT: a novel method for rapid multiple sequence alignment based on fast Fourier transform. Nucleic Acids Res..

[48] Thompson JD, Higgins DG, Gibson TJ (1994). CLUSTAL W: improving the sensitivity of progressive multiple sequence alignment through sequence weighting, position-specific gap penalties and weight matrix choice. Nucleic Acids Res..

[49] Cuff JA, Clamp ME, Siddiqui AS, Finlay M, Barton GJ (1998). JPred: a consensus secondary structure prediction server. Bioinformatics.

[50] Nguyen LT, Schmidt HA, von Haeseler A, Minh BQ (2015). IQ-TREE: a fast and effective stochastic algorithm for estimating maximum-likelihood phylogenies. Mol. Biol. Evol..

[51] Kalyaanamoorthy S, Minh BQ, Wong TKF, von Haeseler A, Jermiin LS (2017). ModelFinder: fast model selection for accurate phylogenetic estimates. Nat. Methods.

[52] Minh BQ, Nguyen MA, von Haeseler A (2013). Ultrafast approximation for phylogenetic bootstrap. Mol. Biol. Evol..

[53] Ran FA (2013). Genome engineering using the CRISPR-Cas9 system. Nat. Protoc..

[54] Concordet JP, Haeussler M (2018). CRISPOR: intuitive guide selection for CRISPR/Cas9 genome editing experiments and screens. Nucleic Acids Res.

[55] Liu Z (2020). Super-resolution microscopy and FIB-SEM imaging reveal parental centriole-derived, hybrid cilium in mammalian multiciliated cells. Dev. Cell.

[56] Liu Z (2020). A quantitative super-resolution imaging toolbox for diagnosis of motile ciliopathies. Sci. Transl. Med..

[57] Mi H, Muruganujan A, Casagrande JT, Thomas PD (2013). Large-scale gene function analysis with the PANTHER classification system. Nat. Protoc..

