# Supplementary material for: Uncovering structural themes across cilia microtubule inner proteins with implications for human cilia function
Source: Nat Commun. 2024 Mar 27;15:2687. doi: 10.1038/s41467-024-46737-3 (PMC10973386; doi:10.1038/s41467-024-46737-3)
Supplement: Supplementary file 1 — Supplementary Information [file 41467_2024_46737_MOESM1_ESM.pdf]

Supplementary Information

Uncovering structural themes across cilia microtubule inner proteins with implications for human cilia function

Jens S. Andersen[1*], Aaran Vijayakumaran[2,6], Christopher Godbehere[2,6], Esben Lorentzen[3], Vito Mennella[2,4], and Kenneth Bødtker Schou[5*]

[1]Department of Biochemistry and Molecular Biology, University of Southern Denmark, Campusvej 55, 5230, Odense M, Denmark. [2]MRC toxicology unit, School of Biological Sciences, University of Cambridge, Gleeson building, Tennis court road, CB2 1QR, Cambridge, United Kingdom. [3]Department of Molecular Biology and Genetics, Aarhus University, Universitetsbyen 81, 8000 Aarhus C, Denmark. [4]Department of Pathology, School of Biological Sciences, University of Cambridge, 10 Tennis court road, CB2 1QP, Cambridge, United Kingdom. [5]The Danish Cancer Society Research Center, Danish Cancer Institute, Strandboulevarden 49, 2100 Copenhagen, Denmark.

[6]These authors contributed equally: Aaran Vijayakumaran, Christopher Godbehere

*Correspondence and requests for materials should be addressed to J.S.A. or K.B.S.

e-mails: jens.andersen@bmb.sdu.dk, kensch@cancer.dk

Supplementary Table S1. Summary of structural properties of human and *C. reinhardtii* microtubule inner proteins (MIPs)

DOMAIN	MIP	*C. Reinhardtii* paralogs	Closest human ortholog	Query	Full length IDP %	Coiled-coil propencity	Sequence match region/boundary	Evidence for MIP functions
NWE	FAP95	FAP68,FAP95, FAP143 FAP107. Note: conserved motif in FAP161	C11ORF1*		31	No	CFAP95:<br>41-193	C11ORF1 (CFAP68) (PMID: 37327785, 36191189)
	FAP107		C1ORF158 (CFAP107)		52	No	CFAP107:<br>18-118	
	FAP143		SPAG8		38	No	SPAG8:<br>272-402	
	FAP161		CFAP161		20	Yes (C-terminus)		
	FAP68	FAP107, FAP143, FAP95	C11ORF1*		26	No	C11ORF1:<br>40-150	
Mn repeat	FAP363	FAP203, FAP257, FAP236	SAXO1/2, MAP6, TEX45, TEX26, MAP6D1, C2ORF73, MDM1		24	No	TEX45:<br>Mn (41-53)<br>Mn (80-92)<br>Mn (170-182)<br>Mn (246-258)<br>Mn (287-293)<br>Mn (408-423)<br>Mn (449-461)<br>TEX26:<br>Mn (31-43)<br>Mn (180-192)<br>Mn (234-244)<br>C2ORF73:<br>Mn (100-112)<br>Mn (150-162)<br>MDM1:<br>Mn (9-21)<br>Mn (189-201)<br>Mn (232-244)<br>Mn (306-318)	TEX45 (SAXO5) (PMID: 37327785)<br>TEX26 (PMID: 37327785).<br>MDM1 (PMID: 26337392)
	FAP273	No			47	No	FAP273:<br>Mn (13-45)<br>RIB21:<br>Mn (123-155)<br>FAP129:<br>Mn1 (212-241)<br>Mn2 (381-413)<br>FAP166:<br>Mn (36-68)<br>SPMIP3:<br>Mn (95-101)<br>SMRP1/SPMIP6:<br>Mn (13-19)<br>TEX37/SPMIP9:<br>Mn (19-25)<br>Mn (104-110)<br>TEX36:<br>Mn (123-129)<br>C3ORF84:<br>Mn (108-114)<br>Mn (140-146)	FAP273 (PMID: 37327785)
	RIB21							RIB21 (PMID: 37327785)
	FAP129							FAP129 (PMID: 37327785)
	FAP166	C-term. EF-hand domain			20	No		FAP166 (PMID: 37327785)
SNYG	FAP85	FAM183A	C5ORF49, FAM183A, ATP6V1FNB		29	No	C5ORF49<br>SNYG (43-127)<br>ATP6V1FNB<br>SNYG (105-163)<br>FAM183A_Hs:<br>SNYG (52-144)<br>FAM183A_Cr<br>SNYG (70-128)<br>FAP85:<br>SNYG (119-197)<br>XP_001699232.1<br>SNYG (133-169)	C5ORF49 (CFAP90) (PMID: 36191189)<br>FAM183A (PMID: 36191189)<br>ATP6V1FNB (SPMIP1) (PMID: 37327785)<br>C20ORF85 (CIMIP1) (PMID: 37327785)<br>TEX49 (PMID: 37327785. 37295417)
	FAM183A	FAP90, FAP85,	C5ORF49, FAM183A, C20ORF85, C2ORF50, ATP6V1FNB, TEX49, SPMIP11_like		15	No	FAM183A:<br>SNYG (9-115).<br>C20ORF85:<br>SNYG (4-124).<br>C2ORF50:<br>SNYG (61-132).	
	FAP182		PIERCE1, PIERCE2				PIERCE1:<br>SNYG (57-117).<br>PIERCE2:<br>SNYG (53-111).	

Supplementary Table S1 (continued). Summary of structural properties of human and *C. reinhardtii* microtubule inner proteins (MIPs)

DOMAIN	MIP	C.Reinhardtii paralogs	Closest human ortholog?	New human ortholog	IDP %	Coiled-coil propencity	Sequence match region	Evidence for MIP functions
PYG	FAP129	PYG motifs in FAP129, FAP222, XP_001689677, and FAP252	FAM166A<br>FAM166B<br>FAM166C<br>C10RF82<br>SPATA48		22	No	C10ORF82:<br>PYG1 (13-65)<br>PYG2 (100-151)<br>PYG3 (171-214)<br>FAM166A:<br>PYG1 (13-51)<br>PYG2 (114-188)<br>PYG3 (213-255)<br>PYG4 (273-317)<br>FAM166B:<br>PYG1 (10-53)<br>PYG2 (87-140)<br>PYG3 (176-215)<br>PYG4 (232-275)<br>FAM166C:<br>PYG1 (19-60)<br>FAP129:<br>PYG1 (25-44)<br>PYG2 (319-359)<br>PYG3 (361-406)<br>FAP222:<br>(55-71)<br>FAP252:<br>PYG (13-34)	FAM166A, FAM166C (PMID: 37327785, 37061538, 36191189).<br>C10ORF82 (SPMIP5) (PMID: 37327785)<br>SPATA48 (SPMIP7) (PMID: 37327785)
	FAP252				22	No		
	FAP222				34	No		
ELLEn	FAP53	FAP53, FAP141	CFAP53<br>CFAP141<br>TCHP		29	Yes	CFAP53:<br>(5-80)<br>TCHP:<br>(20-105)<br>CFAP141<br>(14-92)<br>FAP53<br>(12-86)<br>FAP141<br>(8-102)	
	FAP141				49	No		
						Yes		
GFG repeat	FAP21	FAP77<br>FAP21	EFHB		55	No	EFHB:<br>GFG1 (263-301)<br>GFG2 (302-364)<br>GFG3 (365-420)<br>GFG4 (430-493)<br>GFG5 (495-546)<br>CFAP77:<br>GFG1 (103-140)<br>GFG2 (149-246)<br>FAP21<br>GFG1 (63-120)<br>GFG2 (121-156)<br>GFG3 (157-223)<br>GFG4 (224-282)<br>FAP77:<br>GFP1 (36-72)<br>GFP2 (95-170)	CFAP77 (PMID: 37327785, 37061538, 36191189)
	FAP77		CFAP77					
DM10	FAP67, RIB72	FAP67, RIB72, XP_001691061, DM10DCP	EFHC1, EFHC2, NME7, CAPS2			No	CAPS2:<br>DM10<br>(293-400)	
	FAP20	No	WDR90, CFAP20, CFAP20DC		17	No		
	FAP127	No			52	Yes		
	RIB43a	No	RIBC1, RIBC2		45	Yes		
	FAP112	No			34	C-terminus		
	FAP210	No	No		45	Yes		

Supplementary Table S1 (continued). Summary of structural properties of human and *C. reinhardtii* microtubule inner proteins (MIPs)

DOMAIN	MIP	C.Reinhardtii paralogs	Closest human ortholog?	New human ortholog	IDP %	Coiled-coil propencity	Sequence match region	Evidence for MIP functions
	FAP45	No	CFAP45		45	Yes		
	RIB30	No	No			No		
	FAP126	No	CFAP126		34	No		
	FAP52	No	CFAP52		6	No		
	FAP106	FBB11	Enkurin, ENKD1			No		
	FAP276	No	No		42	No		
	RIB72	No	Three PH domains and C-term. EF-hand and DM10					
	FAP67	No	NDK. DM10		8			
	FAP115	Two EF-hand domains						
	DC1	DC1, DC2, DC3			70	Yes		
	DC2	DC1, DC2, DC3			30	Yes		
	DC3	DC1, DC2, DC3	EF-hand			N-terminus		

Supplementary Table S1. Summary of structural properties of human and *C. reinhardtii* microtubule inner proteins (MIPs). A summary of primary components occupying the axonemal outer doublets analyzed in this study along with the known modules and those newly detected by us. MIPs are grouped according to the seven uncovered protein module and repeat families: NWE, Mn, SNYG, PYG, ELLEn, GFG and DM10. IDP% specifies the degree of protein disorder in the full-length sequence. Sequence match region specifies the boundaries of the sequence homology region (module or repeat unit) that significantly matches with the protein family members (E>0.01). Evidence for MIP function designates the published evidence for the MIPs functions of each protein in the table.

Supplementary Figure S1

a

Expanded alignment of DM10 domain family

```
a|a|Q_EFHB_Hs           ERFVFIAEWYDP--LL---RRYELLFYPGDGSVEMHDVKN---HR---TFLKRTK------LEDLFIGNKVNV--F----SR----QLVLID-Y-GD
a|c|EFHC1_Hs            QVLRFYAIWDDTD-EC---RTYIIHYYLMDDTVEIREVHERDPFP---LLMNRQRVPKV--AKDFIVGKSLTI--L----GR----TFFIYD-C-DP
a|c|EFHC1_Hs            KVLRYLAVLESPI-KD---RRFVFSYFLATDMISIFEPPVRI-GG---KYLGRTKVVKP--PSDFFIGAVIEV--F----GH----RFIILD-T-DE
a|c|CAPS2_Hs            HKLQFDGRIVSRN-AC---RELIGFFFTHDQSLTIYEYRQFTNVL---PFIQKSIYSHQ--LGDFYVGATLTF--L----SSDTLLKLRITN-I-D-
a|c|NME7_Hs             ERFVFIAEWYDP--LL---RRYELLFYPGDGSVEMHDVKN---HR---TFLKRTKYDN---LEDLFIGNKVNV--F----SR----QLVLIDYG-DQ
a|c|EFHC2_Hs            KILCFFCLWDDSV-DR---RELILHYFLCDDTIEIKELLPHDALK---MFLRRSK------DSDLSLGVTINV--W----GR----KVLLYD-C-DE
a|b|EFHC1_1_Dm          YVLRFGAKMLSTI-CE---RIFVISYYLCDDTLQIQEIAVRL-GG---EFMKRTRLELP--PWNFFVGSTMSL--K----DF----IFHIVS-A-DE
a|b|EFHC1_2_Dm          CILRFGAKLISAI-GE---RDFVISYFLADDTLQIYETSRRL-GG---EFLKRARVPLP--ANDFYIGRTMTL--K----DH----IFHIVS-A-DE
a|b|Nmdyn-D7_Dm         RRLAFVAEWFHA--II---RTFLITYYVSDKAVEVFDQRN---KR---TFLRRTKIPE---QRDFFVGSKINV--F----GR----QFDIVD-YADD
a|a|NP_509738_Ce        PMLVYRCCWLDHM-GCRMKRIFKMYVYCSDHTVALIEETKE--GQ---LFLKRIALPFP--WWDIRPGVWVDV--F----CR----PMFVFE-C-A-
a|a|XP_001698964_Cr     AVLRFLASWDNTA-EV---LCFSLFYYVADGTVEVREVLQRDPFP---LLLARGRLPKT--WRDLKLGAKVNC--Y----GR----PLLLYD-C-DE
a|a|XP_001701977_Cr     KVLRFWCVWDERT-DR---RPYVLHYYLEDDSVEVLEINENDPFP---VFLKRGPLPKV--AGDFRLGLFINV--L----GR----DFYLHD-A-DT
a|a|XP_001702841_Cr     LRYCFISEWLDP--IL---WKYQLFYYPESKEVEMVDIKN---RR---HFLKRTKYEE---PSLLFLGSVVTV--F----SR----QLKLTE-YGDE
a|b|XP_001704987_Gl     LCLRFLAKFAPC--AD---RQFVISYFFSDGTVSVFEPTETF-GY---KFLERSRIVNE--FTDLDVGKVVVL--N----AH----CFEIVE-A-DE
a|b|XP_001706258_Gl     TRYSFNVLWYDR--QD---RPYILSYYPDTREIDMYEVAT---KR---VFLKKCQYPE---FADCHVGGTVTI--Y----SR----QLKIVG-YAND
a|c|XP_001770698_Pp     KVLRFFCVWDDRA-ER---RPYVLHYFLGDDTIEILELHERMNFP---MLIKRQRISKE--DTDLHVGSYIRV--F----DR----DLLLHD-V-DE
a|d|XP_001011108_Tt     KVLSFNVLWNDTS-GL---NYYILNYFLADDTTEIKEIKRHDAFP---LFLCRKKLPKE--PSDLVCGNMVRI--Y----NK----DCLIFN-C-DA
a|d|XP_001032591_Tt     QVLRFNAYFKES--RV---RKVVLFFYLEDSSIEISEPKQMP-QG---SFLKRQKVLKA--LYDFQIGQDLEF--F----GK----IFHIYD-C-DI
a|d|XP_012651451_Tt     IRYIFIVEWFDT--LI---RTYYLTYFTQDKTIEMYDLKN---KK---VFLKRCEYA----DSDLYIGSILNV--Y----SR----QLKIVDFA-DV
a|d|XP_001298124_Tv     QVLRFYGYFKEP--RV---RKVKILFFLEDDTIEIVEPRVKP-QG---TFLRRHRAKKD--VGDFHIGEEFSL--Y----GR----VYFIQE-A-DK
a|d|XP_001300615_Tv     HVLRFYATWDNRD-DL---RQFVIHYYLSDDTMEVLEVRHQDPVP---SFVKRGQIPKR--PTDLHIGDDIQI--L----GR----TMHIFD-C-DP
a|d|XP_001303316_Tv     TVLSFKSHLVSNN-IL---RQFIVNYFVDDDKFSVFEKVVPP-GG---NFISRAKFINP--TEELYIGATVVL--N----SW----TFQLDE-A-TE
a|d|XP_001320748_Tv     PKYAFALQYFDT--LL---RDYIIQFYTERGEIDIYDCRA---KR---MILRKTLEHK---LSDLYVGNKILV--N----GR----QYDIVD-Y-A-
a|d|XP_001323370_Tv     PTYSFKLEFYDE--LV---RPYVLMYFTHLGEIEIYDLRQ---KR---IFLKKTTNHN---IKDLYIGNKLLI--N----GR----QYEIIE-YQNE
a|d|XP_001324030_Tv     QVLNFKAKMLSDY-RR---RQFMISYYLCDKTMAIFEANVPR-AG---KFLQRTRVRNP--PEAFYVGAKIQA--S----GR----VFELLD-A-AP
a|d|XP_001326363_Tv     TVLSFKSHLVSNS-IL---RQFIVNYFVDDDKFSVFEKVVPP-GG---NFISRAKFINP--TEELYIGATVVL--N----SW----TFQLDE-A-TE
a|d|XP_001328751_Tv     GVLRFYAYFREE--RI---RYVRIYAYLEDNTIMIEEQKQRD-QG---VLLKRMRALKP--AADFNVGQNYEI--Y----GI----TYHIYA-C-DE
a|d|XP_001329899_Tv     AKVTFQSKLITPN-KQ---RIFLLTYFVNTKEISIQEGRSP--AH---PYYSRGKITNP--PKDFYVGSTITA--L----GR----KFYLTD-A-SQ
a|d|XP_001579855_Tv     TILQFSATIISDN-KL---RSFVVQYYVEDKAFQVFEKVVPN-GG---KFITKTVCNNP--PKDIFLGAKVNI--N----GF----RFILQE-A-SE
a|d|XP_001580450_Tv     YTLRFYGYFQQS--RI---RYVNIYVYTEDDTVMIEEQKSRT-QG---VLLRRMRVQNP--VQDFQIGVNIDI--S----GI----VYHIYD-C-DQ
a|d|XP_001581058_Tv     NELRFLAKMISKN-DK---REFSVIFSLVNDEVKVWENKTD--GG---FVYKAPH----PHYNDMYIGANVEI--N----HV----VYKLYG-A-PE
a|d|XP_001583309_Tv     ENLVFNAKMITEN-KA---RVFSISFNLREKKLSILEGKSS--PQ---RFLSPSTVIDP--ESSFYIGSRIIA--A----GR----LFELVD-A-SD
a|d|XP_001584321_Tv     LVLRFYAYFREN-YRV---RYVKIMVYLEDDTVMIEENHVRA-QG---VLLRRMPVLNP--NLDFKVGINIEI--F----GI----VYRIYA-C-DQ
a|d|XP_002365869_Tg     KVLRFYCFWDDKT-MR---SYYVIHYFLADDTVEILECYPKREFP---TFLKRQQLPKE--PEDFEVGKEITV--Y----NR----QFHLYD-C-DE
a|d|XP_018636946_Tg     MVCRFFASYDDSL-ER---RLMVILFYLCDDTIEIREHLPTPHFP---VYFRRRKLPKT--ITDFKVGSMMRM--L----DV----DFFIYD-A-DG
a|d|XP_018637413_Tg     DTWVFHVTWCDH--MV---KELSLSYNRSDNSVELYDPKL---RR---LFLKRTPASI---EGHLYLGNTVTI--F----SR----QLKIVD-YGDE
b|XP_001424952_Pt       AVLRFYSYFQES--RI---RQVIIYYYLSDGTIHVTEPRVQP-QG---LFIKRQKIPKK--WDDLQLCSNINF--Y----DR----VYRICD-C-DT
b|XP_001425784_Pt       ERYVFIVEWFDT--LI---RSYNLIYFMADKTIEMFDLKN---KR---IFLKRCDYPS---LKDLYVGSIVTV--F----SR----QLKIVD-YADV
b|XP_001426105_Pt       HVLRFSGYFKEA--RI---RKITICYYLEDHSLSITEPKQEP-QG---AFLKRQKVLRA--PEDFRINQDIII--F----GK----TIRLYD-C-DQ
b|XP_001426356_Pt       HVLRFSGYFKEA--RI---RKLTIFYYLEDHSLSITEPKQEP-QG---AFLKRQKVLRA--PEDFRINQDIEI--F----GK----TIRLFD-C-DQ
b|XP_001428401_Pt       KVLSFDIIWDDAS-QL---NYYTLNYYLADDTCEVKEVRKQDPYP---LMLRRQKIPKQ--PQDLICGNQVRI--Y----GR----DCFIYG-C-DD
b|XP_001431229_Pt       KVLSFDVIWDDAS-QL---NYYTLNYYLADDTCEVKEVRKQDPYP---LMLRRQKIPKT--PQDLICGNTVKI--Y----GR----ECFIYG-C-DD
b|XP_001432060_Pt       KVLKFYVFSD---------IEYVLHYYLADDTIEIKEINSAVPFP---MMLRRQKLPRK--PQDIQFGQPLII--Y----NR----KFAIQG-C-DP
b|XP_001435489_Pt       HVLRFSGFFKEA--RI---RKLTIFYYLEDHSISITEPKQEP-QG---AFLKRQKVLRA--PEDFRINQDIEI--F----GK----TIRLYD-C-DQ
b|XP_001439127_Pt       HVLRFSGYFKEA--RI---RKITLFYYLEDHSLSITEPKQEP-QG---AFLKRQKVLRA--PEDFRINRDIII--F----GK----TIRLFD-C-DQ
b|XP_001442446_Pt       ERYVFIVEWFDT--LI---RSYNLTYFMSDKTIEMFDLKN---KR---IFLKRCEYPS---LKDLYVGSIVTV--F----SR----QLKIVD-YADV
b|XP_001450194_Pt       MFYEFSGYFKEAV-RI---RKITLFYYLEDHSLSITEPKQEP-QG---AFLKRQKVLRA--PEDFRINRDIII--F----GK----TIRLFD-C-DQ
b|XP_001451738_Pt       HVLRFSGYFKEA--RI---RKITIFYYLEDHSLSITEPKQEP-QG---AFLKRQKVLRA--PEDFRINQDIII--F----GK----TIRLFD-C-DQ
b|XP_001452778_Pt       ERYVFIVEWFDT--LI---RSYNLTYFMSDKTIEMFDLKN---KR---IFLKRCEYPS---LKDLYVGSIVTV--F----SR----QLKIVD-YADV
b|XP_001455501_Pt       ERYVFIVEWFDT--LI---RSYNLIYFMADKTIEMFDLKN---KR---IFLKRCEYPS---LKDLYVGSIVTV--F----SR----QLKIVD-YADV
b|XP_001458578_Pt       KVLSFDIIWDDAS-QL---NYYTLNYYLADDTCEVKEVRKQDPYP---LMLRRQKIPKA--PQDLICGNTVKI--Y----GR----ECFIYG-C-DD
b|XP_001459424_Pt       KVLKFYVFSD---------IEYILHYYLADDTIEIKEINSAVPFP---MMLRRQKLPRK--PQDIQFGQPLII--Y----NR----KFLITG-C-DQ
b|XP_011772031_Tb       EHLTFLALWDDRE-DL---RFVVIRLYLENNTVEIIERRQEMGSS---VILGRQRVARP--AEDMKVGETYHI--H----GR----PYFIYD-A-DE
b|XP_011772748_Tb       PRLSFYCEQYDH--RM---NHYVLQFYFEDRTVEIREVTK---NR---LHLKRAHFPH---RDDFKVGSSLSL--L----GG----VIKLTA-YADE
b|XP_011773562_Tb       KVLRFYGLLDER--AV---RKLEVLYFVEDDSIAVVERPTT--PA---LFLSRGW------ARDLGVGATINV--L----GR----GVFLYD-C-DD
b|XP_011774056_Tb       DSLLFHCVQLSQG-SH---RYFFGCYFPRWRGFYMDEAREL--GYNVFPAFPFDVYLKD--TDDFQIGSIFTL--GGPDGQK----RYKVVH-C-DD
b|XP_011776862_Tb       ERYAFKVDYCDP--LV---RQYLLLYFAEDSTIEMHDLKT---KR---VFLKRCAYPS---PRELFIGATVGV--F----SR----SLKLVD-YGDE
b|XP_011778115_Tb       EVLRFAATWE-----Q---RAFKLLYYIADKTMSVMVESVRDPNP---VFIRRTKIPKY--EDDLQTGQTINL--M----TR----EFYIYD-C-DK
b|XP_011779300_Tb       EVLRCDCVWDDME-TK---HYLTLYYFLSDDSIALVEKDYPDPFP---RFFRRQRVAKP--DADIRIGNCLHV--F----GR----DVLIYD-Y-DE
```

b

RPE-1 cells

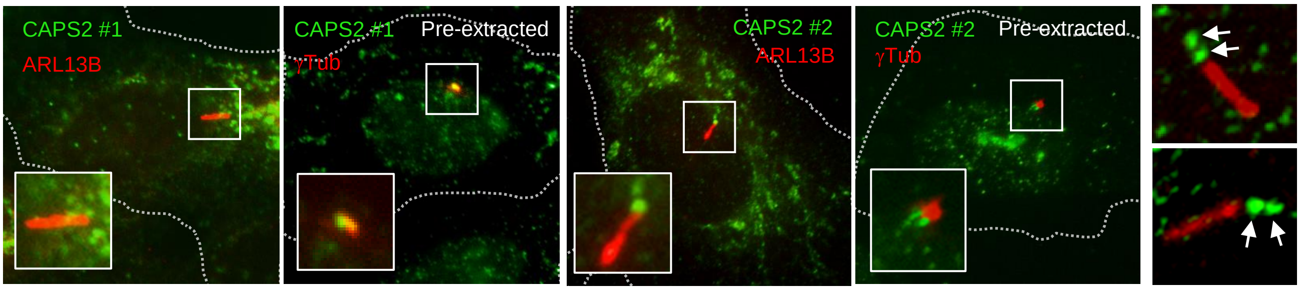

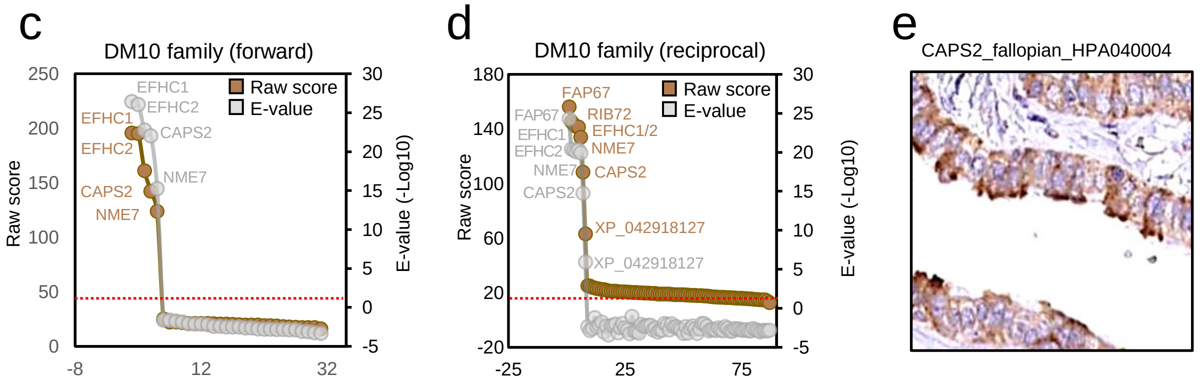

Supplementary Figure S1

The DM10 domain in CAPS2 is highly conserved and CAPS2 localizes to the base of primary cilia. **a** Expanded sequence alignment of the DM10 domain family across many species. Species represented are *Homo sapiens* (Hs), *Drosophila melanogaster* (Dm), *C. elegans* (Ce), *Chlamodomonas reinhardtii* (Cr), *Giardia lamblia* (Gl), *Physcomitrella patens* (Pp), *Tetrahymena thermophila* (Tt), *Trichomonas vaginalis* (Tv), *Paramecium tetraurelia* (Pt), and *Toxoplasma gondii* (Tg). **b** Left, Immunofluorescence microscopy images of serum-starved hTERT RPE1 cells co-stained with CAPS2 #1 (ProteinTech) and ARL13B after PFA fixation or CAPS2 #1 (ProteinTech) and γ–tubulin in cells pre-extrated with CSK buffer prior to PFA fixation. Right, immunofluorescence microscopy images of serum-starved hTERT RPE1 cells co-stained with CAPS2 #2 (HPA) and ARL13B after PFA fixation or CAPS2 #2 (HPA) and γ–tubulin in cells pre-extrated with CSK buffer prior to PFA fixation. Arrows indicate both centrioles stained by the CAPS2 #2 antibody. Scale bar: 10 μm. Images are representative of X=2 independent experiments. **c**. Probability plot of the DM10 domain searches using DM10 from human EFHB1 as a search query (forward search). Only human proteins are shown. The dashed red line indicates the E = 0.01 threshold value. **d** Probability plot of the DM10 domain searches using the DM10 from *C. reinhardtii* FAP67 as a search query (reciprocal search). Both human and *C. reinhardtii* proteins are shown. The dashed red line indicates the E = 0.01 threshold value. **e** Fallopian tube tissue staining using the CAPS2 antibody (HPA040004). Image obtained from The Human Protein Atlas (https://www.proteinatlas.org/).

Supplementary Figure S2

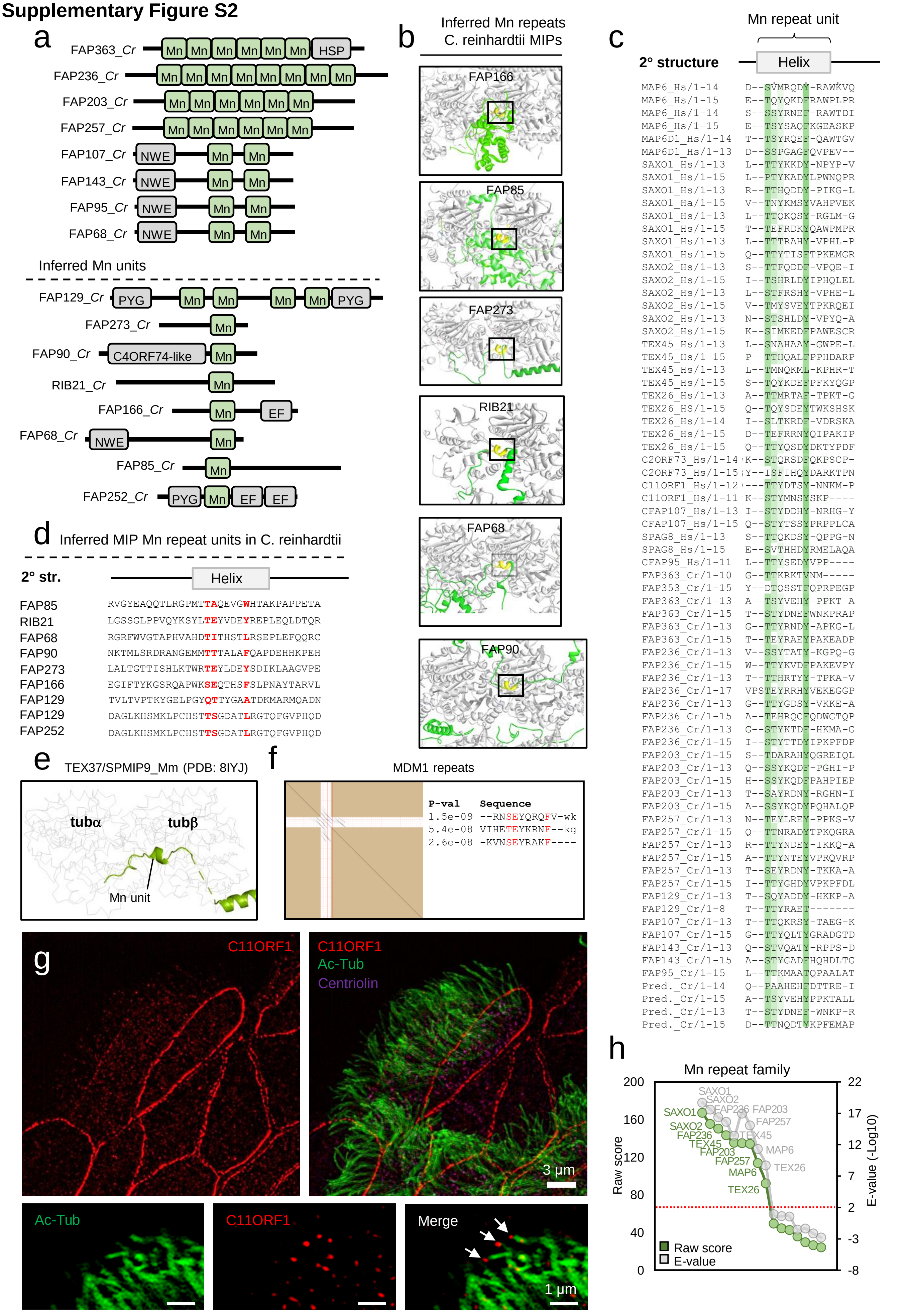

Supplementary Figure S2

Mn units in human MIPs and inferred Mn repeats in *C. reinhardtii* MIPs. **a** Architecture of Mn repeat-containing proteins from *C. reinhardtii*. **b** Mn repeat units of MIPs bound to the MT lattice in the outer doublet as identified in PDB: 6U42. The MIPs are highlighted in green and individual Mn repeat units are highlighted in yellow. **c** Expanded alignment of Mn repeat units from human and *C. reinhardtii* MIPs. Coloring schemes as per ClustalX parameters with conserved residues highlighted in green. Predicted secondary structure of the aligned Mn repeat units are indicated above the alignment. **d** Inferred Mn repeat unit sequences aligned according to the MN repeat unit signature sequence highlighted in red. **e** The Mn repeat unit of TEX37 (SPMIP9) in complex with the MT lattice. Coordinates were retrieved from PDB: 8IYJ and analyzed in PyMol. **f** HHrepIP protein repeat analysis results of MDM1 **g** Immunofluorescence microscopy micrographs of motile cilia in cultured human bronchial epithelial cells. Cells were stained with indicated antibodies. Arrows indicate the cilia tip enrichment of C11ORF1. Images are representative of X=2 independent experiments. **h** Probability plot of the Mn repeats searches using human TEX45 as a search query (forward search). The dashed red line indicates the E = 0.01 threshold value. Both human and *C. reinhardtii* proteins are shown.

Supplementary Figure S3

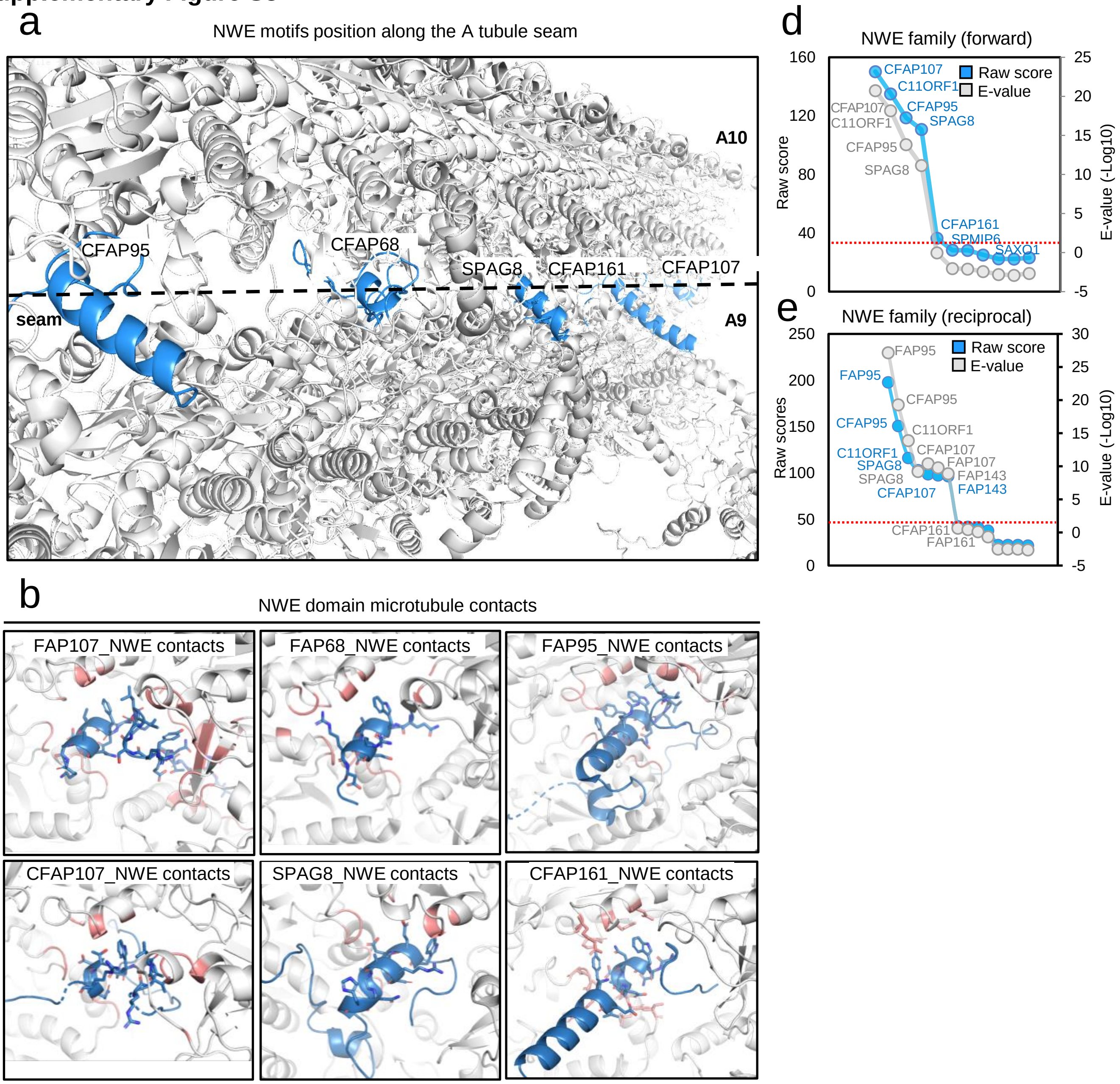

c

Expanded alignment of NWE module family

```
a|CFAP68_Hs          NTY---SNRTLMGNWNQERY-D---LRNQFGHY----FETTYD---TSYNN------KRFKR--EPHWFP---GHQP---ELDPPRY-KCTEKSTYMNSYSK---
a|SPAG8_Hs           --P---RGQCLLYNWEEERA-----TNHKSPMP----SSTTQK---DSYQP-------QIC----KEVQA---EQEP---T----RK-LFEVESVTHHDYRM---
b|Q_CFAP107_Hs       TKY---STKVLTGNWMEERR-----FTR----T----PQSIYR---KEYIP--KRKVEGLP----YKHLI---THHQ---E----PP-HRYLISTYDDHYNRHG-
b|CFAP95_Hs          -KY---SKPVLVYSWHRDREA----LGT----I----WTSETH---EKLSQ--LLNEETVSS--FGAVFP---RHPP---D----WS-KMCALTTYSEDYVPP--
a|CFAP68_Mm           NSY---SNRTLVGNWNQERY-D---LKNQFGHA----FETTYD---ANYSR-------THRFKREPHWFP---GHQP---ELDPPHY-KCTAKSTYMTNYSEP--
a|CFAP107_Mm         -KY---STKVLLGNWVEERG-K---FTK--DHT----PQCIYR---KEYVP-------GLP----YKHLI---THHQ---E----PS-HRYLISTYDDHYNR---
a|CFAP95_Mm          -KY---SEPVLVYSWHRNRE-A---FPKESLET----VKNLCM---STYKR-------TLC----PGILERPMESPP---D----PR-KPCFSTTYSEDFTP---
a|SPAG8_Mm           --P---RGQCLLYNWEEERA-----TNQQSPMS----SSTTQR---DSYQL-------QIC----KEVQA---EQEP---A----RK-LFETESVTHHDYR----
a|F1NTY2_Gg          -NY---SHKVLMGNWNEERY-D---IQNQYAHC----FETTYS---SDYNK-------TRRFEQEPHWFP---GHQP---ELEPPLF-KSTAQSCYTIDYRPP--
a|F1P4Y1_Gg          -KY---SPKVLIGNWLEERK-R---FIKGCSIY----STDFTHFPERTLRT------IGLP----AQHFF---THHE---E----PR-SRHLVSEYEDNYNR---
a|A0A6I8RD92_Xt      NSY---SNKTLMGNWNQEHF-D---LSHQYDHY----YQSSYS---SSYNK-------GFKR--EPHIFP---GHQP---ELESLES-KPLQKSCYMLDYCHP--
a|F6ZUW5_Xt          -KY---SDKVLIGNWVEERR-K---FRT----R----YQPKQD---SCYNI-------GLP----KCHLL---SHHG---E----PK-HKHLVSLYDDHY-----
a|A0A803K5R1_Xt      -TY---GRPTLVSGWHQNRE-A---EPKTLPVG----KKNLCQ---STYRS-------SQR----GYVLP---QHDK---D----HG-KMHLKTTYYIDYS----
a|A0A803J3Y5_Xt      ------RSPCLLSNWVEERA-----TASLAKLM----DSTTYR---DSYTQ-------KFR----KEILS---EINS---S---AQE-PTLTESTTKRDYQ----
a|F6ZUW5_Xt          -----------------RE-Q---FSE----D----HKSLYS---ASYKQ-------TVRFGVAPRVLS----------ST-MYPS-NNNNKSMNFREYR----
a|A0A0G2L2X8_Dr      -LY---SNSTLIGNWSEESF-D---ARRQFSHY----FETTYH---SGYKK-------YTRPKREPRGFP---GHQP---ELDPPHT-KCVPDSCYRMDFK----
a|A0A2R8QQP5_Dr      -LY---SNSTLIGNWSEESF-D---ARRQFSHY----FETTYH---SGYKK-------YTRPKREPRGFP---GHQP---E----LD-PPHTK-----------
a|E7F2M2_Dr          -KY---GNKVLIGNWVEEKL-Q---FAR--CKT----ANSSNR---LDYIP-------GVP----SRQLL---SHHD---V----PS-SHYLVSLYDESYG----
b|b|NP_649418_Dm     TPF---GKEVLIGNWAERRY-----VEE----L----HRSLCH---DTYTT--YKNFIRNR----RSSLN---LVDD---E----LL-KRNFTTTNTLEFQ----
b|a|FAP68_Cr         PSY---NAETCMQNWAEDRR----HYKS--STR----YDSEYS---ARFKP-------------------------------------------------------
b|a|FAP107_Cr        NEY---RSGVLIGNFVENAA-----TGR----PG--IPTTTQK---RSYTA--DLNQPGVK----GELLT---RHGRFD-E----PP-VQCLGTTYQLTYGRAD-
b|d|FAP143_Cr        ------EVPTLIGNWVEERE-ELTGVTR----D----WQSTVQ---ATYRPP-EQLMREAQ----DLPPE---LQATLTGP----AV-PVTTASTYGADFHQHD-
b|e|RIB38_Tt         -KY---TSTALMGNWYEERL-----PNQ----E----FRSVNR---TSYDP--LLNQTNSQH--FGSTFK---KFPE---T----HE-KFFGMTTYQQSFSRP--
b|e|CFAP107_Tt       NKY---RFGVLIGNFAEEKF-----MDM----L----PNSTMK---DSYGL--IFNTQGVQ----QHILF---GHGV---K-QTDYN-KREYGTSYDLSFNQKI-
b|e|CFAP143_Tt       NKQLQKGQGTLIGNWWEERE-D--VTGI----HNFNHIPNTTN---SEYGKG-QQMINHIK----QELNE---IENQKEIQ----RN-IRYFQTTTQTEFGPKE-
b|d|XP_001458102_Pt  VEY---SKTALMANWYEDRL-----PQQ----II--EFRTEFQ---TKFDA--QLDQTNMQQ--FGSTFR---NHPE---Q----HN-KFYSITTYQQSFQPP--
b|d|XP_001438573_Pt  NKY---RYGVKIGNYNEETF-----MDV----I----PNSTMK---DTFGL-TTFSHGGLQ----NHLLL---GHGQQ--S----YFETRQLTSTYDLSHNQQV-
b|d|XP_001455231_Pt  NTF---VSGTLMSNWHEEAQ-EDTGFGR----A----SQNVGG---TVNRA--QQYLQQIQ----AELDSREADSFS---Q----TQ-QRYFETTCKQEFTQKP-
b|e|XP_001582275_Tv  NLE---DNKTLIGNWYEERE-----QAAS-KPA----QIDHSEYT-EQTQP--RQAAREIT----RDIQN---EQLA---A------------EDHKMYFTRGKF
b|e|XP_001312332_Tv  --F---GNRTLVGNWWEERQ-----LQE----P----VASTYK---LDTDQ-RKLAMGSTQT---FDTLN---TRFY---D----PN-KTRFKTVYQKSYNIP--
```

Supplementary Figure S3

The NWE module binds to the cilia outer doublet MT seam in mammals. **a** NWE motifs across the cilia outer doulet seam in *human*. NWE-containing MIPs are shown in blue, and the NWE motifs are shown in red boxes (PDB: 7UNG). **b** A-tubule seam contacts with NWE motifs of *C. reinhardtii* MIPs FAP107, FAP68, FAP95, and *B. taurus* CFAP107, SPAG8, and CFAP161. Contacts shown in red were assessed in PyMol. **c** Expanded alignment of NWE module family members across species. Proteins are designated by their UniProt identifiers. Species represented are Homo sapiens (Hs), *Mus musculus* (Mm), *Giargia gamblia* (Gg), *Xenopus tropicalis* (Xt), *Danio rerio* (Dr), *Drosophila melanogaster* (Dm), *Chlamydomonas reinhardtii* (Cr), *Tetrahymena thermophila (*Tt*)*, *Paramecium tetraurelia (*Pt*)*, and *Trichomonas vaginalis (*Tv*)*. Coloring schemes as per ClustalW parameters with modifications. **d** Probability plots of NWE family member search matches using NWE module portion from human CFAP107 as a search query (forward search). Only human proteins are shown. The dashed red line indicates the E = 0.01 threshold value. **e** Probability plots of NWE family member search matches using NWE module portion from *C. reinhardtii* FAP95 as a search query (reciprocal search). Only human and *C. reinhardtii* proteins are shown. The dashed red line indicates the E = 0.01 threshold value.

Supplementary Figure S4

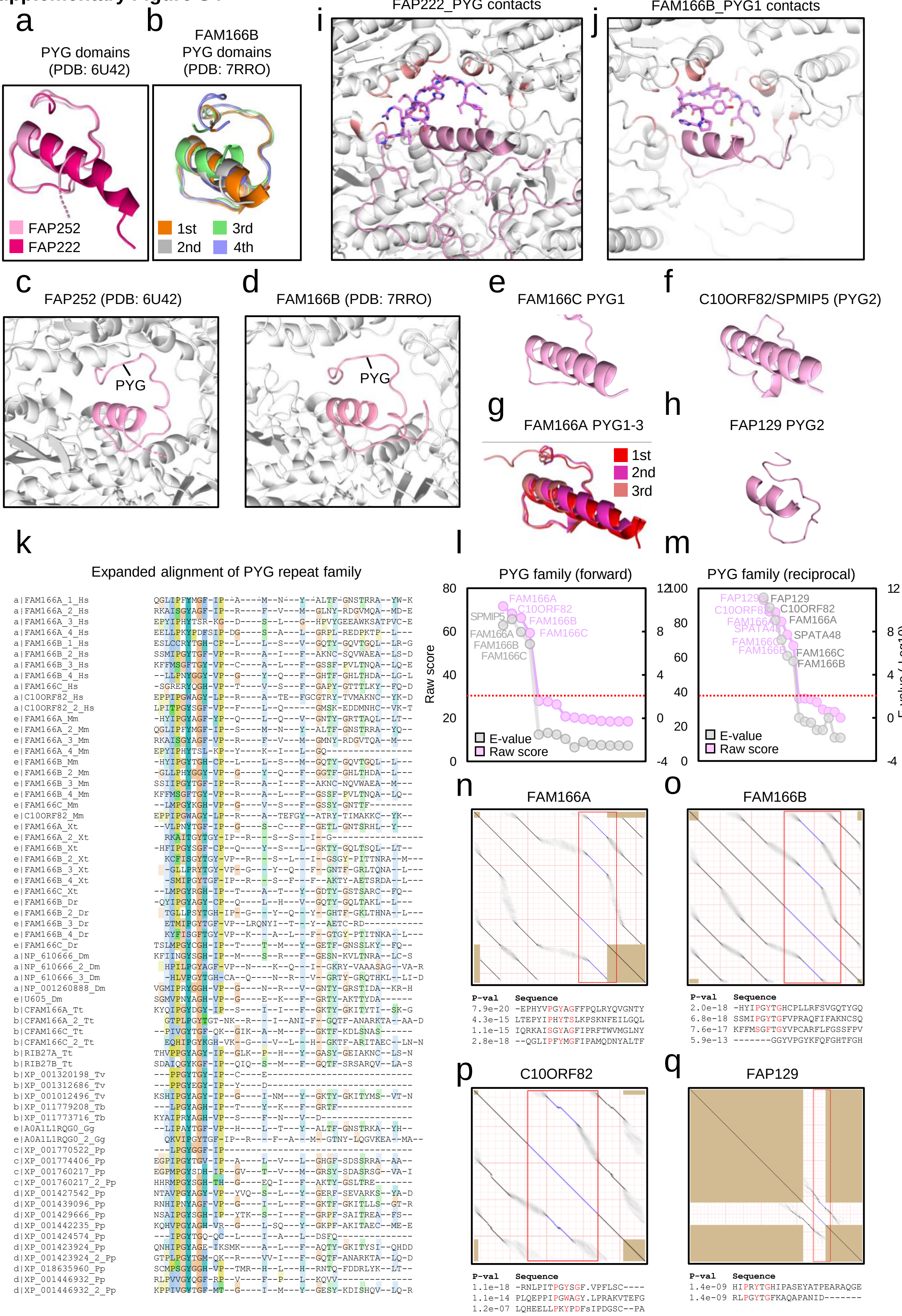

Supplementary Figure S4

The PYG repeat is found among MIPs across species. **a** Superimposition of the PYG repeats found in *C. reinhardtii* FAP22 and FAP252. **b** Superimposition of PYG repeats in *B. taurus* FAM166B. (**c**) The FAP252 PYG repeat unit in complex with tubulins (PDB: 6U42). **d** A PYG repeat unit from FAM166B in complex with tubulins (PDB: 7RRO). **e** AlphaFold2 structural prediction of a PYG repeat unit from FAM166C. **f** AlphaFold2 structural prediction of a PYG repeat unit from C10ORF82 (SPMIP5). **g** AlphaFold2 predictions of three PYG repeat units from FAM166A. Superimpositions was performed in PyMol. **h** A PYG repeat unit from FAP129 (PDB: 8GLV). **i** The FAP222 PYG repeat showing its contact sites with the luminal MT lattice (PDB: 6U42). Contact sites were assessed in PyMol. **j** A PYG repeat unit of FAM166B and its contact sites with the luminal MT lattice (PDB: 7RRO). Contact sites were assessed in PyMol. **k** Expanded alignment of PYG repeats in MIPs across species. Coloring schemes as per ClustalX parameters with modifications. Species represented are *Homo sapiens* (Hs), *Mus musculus* (Mm), *Xenopus tropicalis* (Xt), *Danio rerio* (Dr), *Drosophila melanogaster* (Dm), *Tetrahymena thermophila* (Tt), *Trichomonas vaginalis* (Tv), *Trypanosoma brucei* (Tb), *Giardia glambia* (Gg), *Physcomitrella patens* (Pp). (**l**) Probability plot of the PYG repeat searches using PYG bearing portion from human FAM166A as a search query (forward search). Only human proteins are shown. The dashed red line indicates the E = 0.01 threshold value. **m** Probability plot of PYG repeat searches using the two C-terminal tandem PYG repeats from *C. reinhardtii* FAP129 as a search query (reciprocal search). Both human and *C. reinhardtii* proteins are shown. The dashed red line indicates the E = 0.01 threshold value. **n-q** Protein sequence repeat analysis results as represented by dot plot patterns as assessed by the HHrepID software. For each of the FAM166A, FAM166B, C10ORF82, and FAP129 MIPs, HHrepID detects most of the PYG repeats also identified by profile-HMM searches.

Supplementary Figure S5

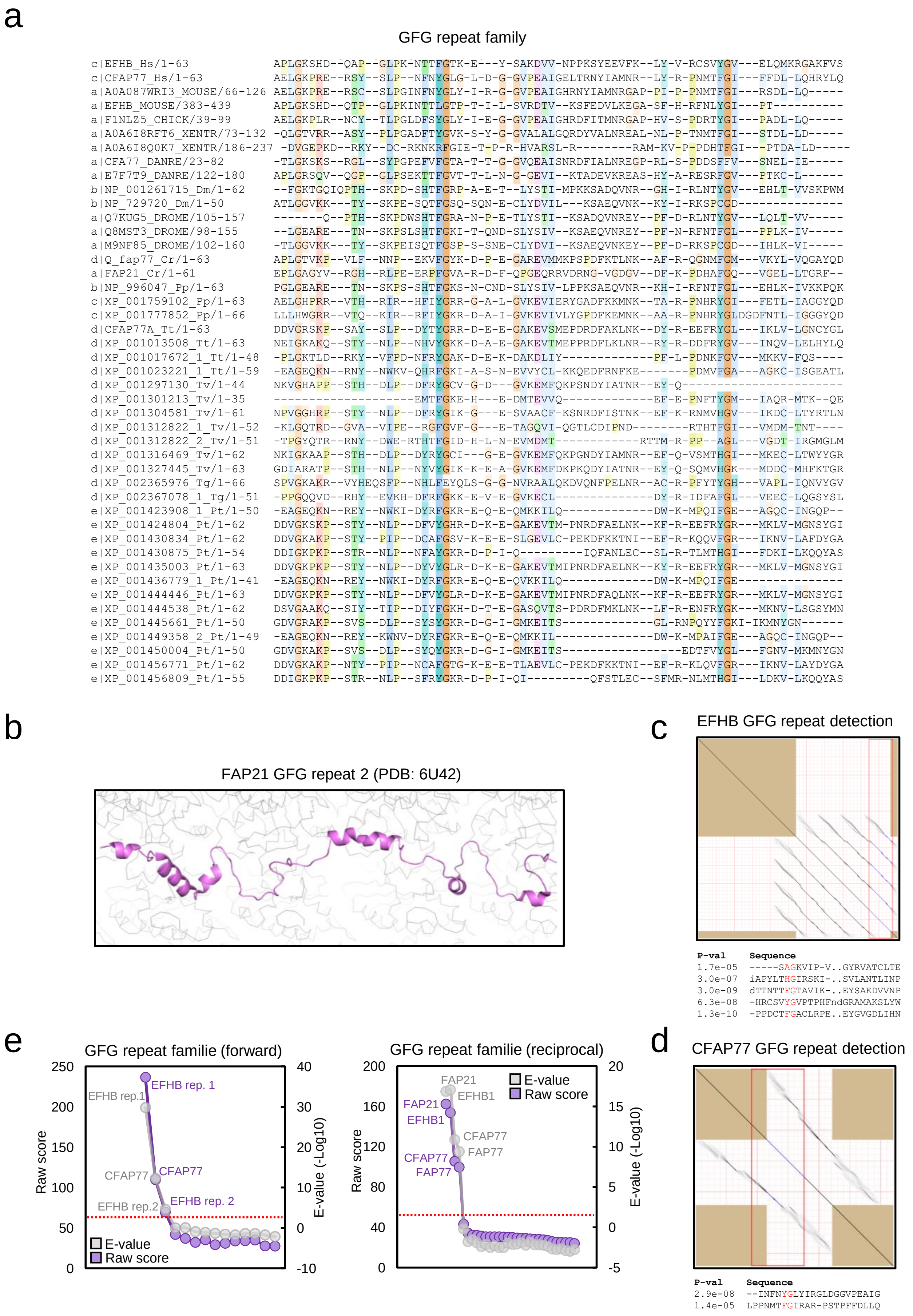

Supplementary Figure S5

The EFHB, FAP21, CFAP77, and FAP77 MIPs bear MT-binding GFG repeats. **a** Expanded alignment of the GFG repeat family of proteins across species. Coloring schemes as per ClustalX parameters with modifications. Species represented are *Homo sapiens* (Hs), *Mus musculus* (Mm), *Gallus gallus* (Gg), *Xenopus tropicalis* (Xt), *Danio rerio* (Dr), *Drosophila melanogaster* (Dm), *Chlamodomonas reinhardtii* (Cr), *Physcomitrella patens* (Pp), *Tetrahymena thermophila* (Tt), *Trichomonas vaginalis* (Tv), *Paramecium tetraurelia* (Pt), and *Toxoplasma gondii* (Tg). **b** A GFG repeat unit of FAP21 in complex with the luminal tubulin lattice (PDB: 6U42). **c**. EFHB protein sequence repeat analysis results as represented by dot plot patterns as assessed by the HHrepID software. HHrepID detects all of the GFG repeats also identified by profile-HMM searches. **d** CFAP77 protein sequence repeat analysis results as represented by dot plot patterns as assessed by the HHrepID software. HHrepID detects both of the GFG repeats also identified by profile-HMM searches. **e** Left, probability plot of GFG repeat searches using a GFG repeat portion from Human EFHB as a search query (forward search). Only human proteins are shown. The dashed red line indicates the E = 0.01 threshold value. Right, probability plot of GFG repeat searches using a GFG repeat portion from Human FAP21 as a search query (reciprocal search). Both *C. reinhardtii* and human proteins are shown. The dashed red line indicates the E = 0.01 threshold value.

Supplementary Figure S6

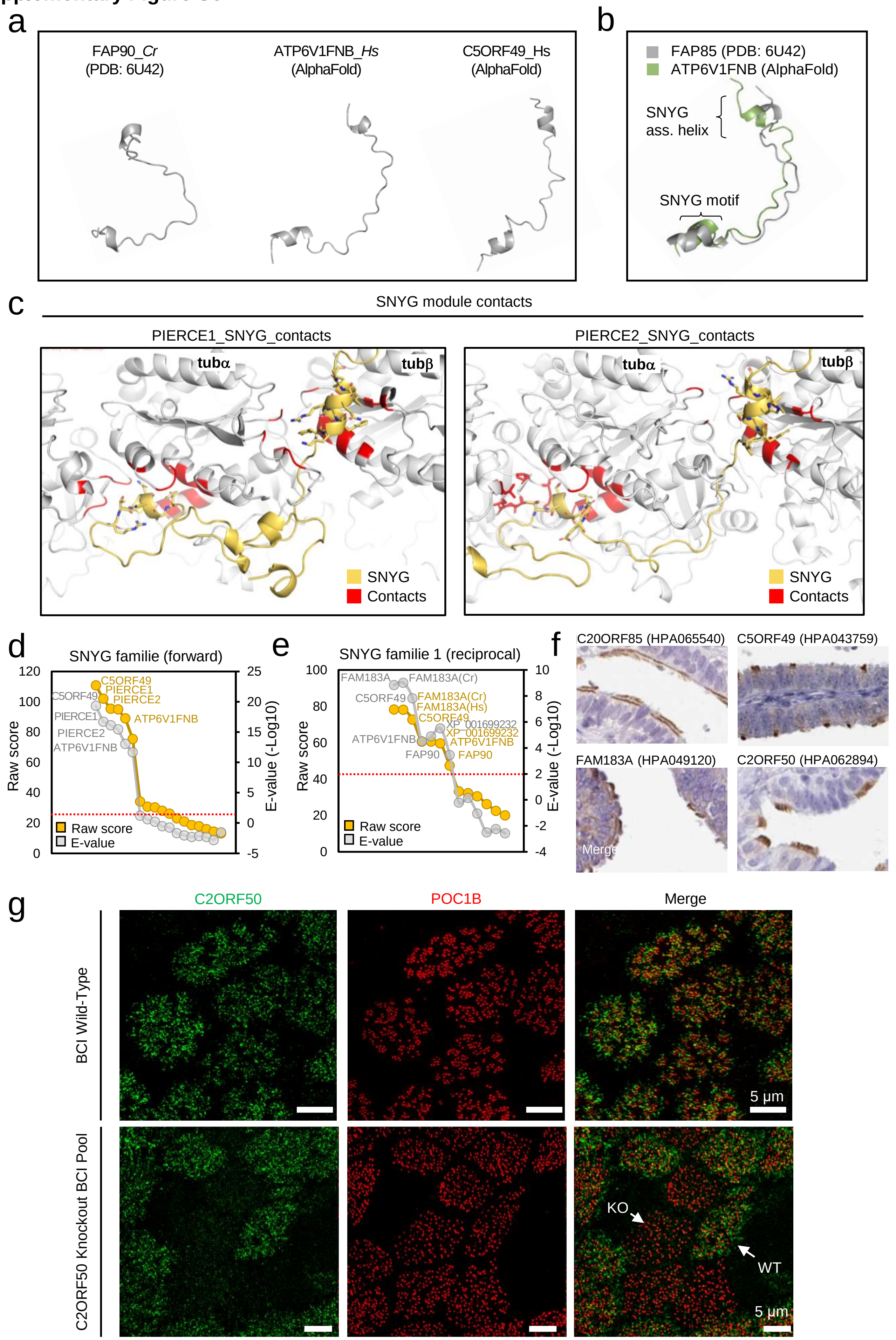

Supplementary Figure S6

Predicted SNYG modules in *C. reinhardtii* and human MIPs. **a, b** Structures of some SNYG modules. ATP6V1FNB and C5ORF49 structures were modelled in AlphaFold2. *C. reinhardtii* FAP85 and FAP90 were retrieved from PDB: 6U42. The SNYG module is structurally similar to the Mn repeats but appear in all cases to be kinked rather than having the elongated SAXO protein family-like structure. This is probably due to the conserved glycine found in SNYG consensus motif (Fig. 7a). **c** The SNYG module contact sites of PIERCE1 and PIERCE2 in the luminal tubulin lattice (PBD: 7RRO). Contacts were assessed in PyMol **d** Probability plot of SNYG family module searches using a SNYG containing region from human C5ORF49 as a search query (forward search). Only human proteins are shown The dashed red line indicates the E = 0.01 threshold value. **e** Probability plot of the SNYG family searches using a SNYG containing portion from *C. reinhardtii* FAM183A as a search query (reciprocal search). Both *C. reinhardtii* and human proteins are shown. The dashed red line indicates the E = 0.01 threshold value. **f** Fallopian tube tissue staining of PIERCE1, C20ORF85, C5ORF49, FAM183A, and C2ORF50. Image obtained from The Human Protein Atlas (https://www.proteinatlas.org/). **g** Immunofluorescence microscopy micrographs of motile cilia in cultured human bronchial epithelial cells from control or knockout cells for C2ORF50 by CRISPR Cas9. Arrows indicate examples of control and knockout cells in the CRISPR Cas9 pool. Cells were labeled with indicated antibodies. Images are representative of X=2 independent experiments.

Supplementary Figure S7

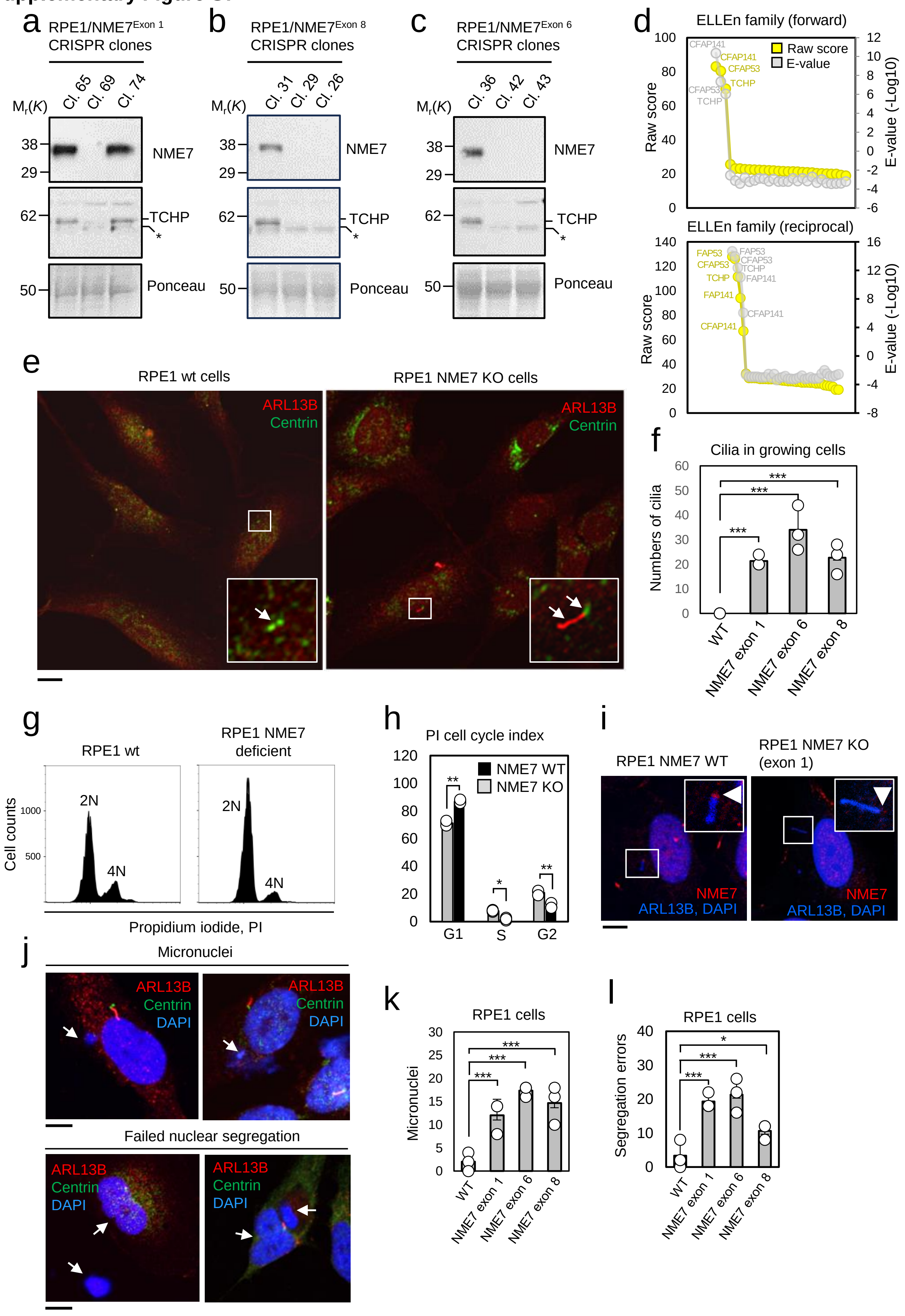

Supplementary Figure S7

The ELLEn module is a universal NME7-binding interface required for cilia or basal body functions of NME7. **a**-**c** Immunoblot results showing NME7 and TCHP protein levels in NME7 WT and CRISPR knockout clones. Three exons in NME7 in RPE1 cells were targeted by CRISPR, exon 1, exon 6, and exon 8. In all cases NME7 is knocked out and TCHP is co-depleted in those same clones. Proteins were probed with the indicated antibodies. Immunoblots shown represents X=2 independent experiments. **d** Upper, probability plot of the ELLEn module searches using a ELLEn module containing region from human CFAP141 as a search query (forward search). Only human proteins are shown. Lower, probability plot of the ELLEN module searches using an ELLEn-containing portion from *C. reinhardtii* FAP53 as a search query (reciprocal search). Both *C. reinhardtii* and human proteins are shown. The dashed red line indicates the E = 0.01 threshold value. **e** Immunofluorescence microscopy images of RPE1 WT or NME7 KO cells. Cells were stained with the indicated antibodies. The shown images are representative of X=3 independent experiments. **f** Quantifications of the premature cilia formation in cycling RPE1 WT or NME7 KO cells. 200 cells were counted per condition in X=3 independent experiments. **g** FACS analysis of cycling RPE1 WT or NME7 KO cells. Cell cycle distribution was measured by propidium iodide staining of DNA content. **h** Quantification of DNA content of FACS PI profiles in (g) based on 30,000 cell counts per experiment. Histograms and statistical analysis are representative of X=3 independent experiments. **i** Immunofluorescence microscopy images of RPE1 WT or NME7 KO cells stained with DAPI and the indicated antibodies. Images are representative of X=3 independent experiments. **j** Immunofluorescence microscopy images of NME7 KO cells showing their deficiencies in chromosome segregation. Cells were stained with DAPI and the indicated antibodies. Images are representative of X=3 independent experiments. **k**, **l** Quantifications of cells with nuclear or chromosome defects in **j**. 200 cells were counted per condition. The quantification represents X=3 independent experiments. Bars represent mean ± s.e.m. P values result from one-sided, one-way ANOVA followed by Bonferroni correction (***, Pr≤0.001; **, Pr≤0.01). Scale bars in e, j, i 10 μm.

Supplementary Figure S8

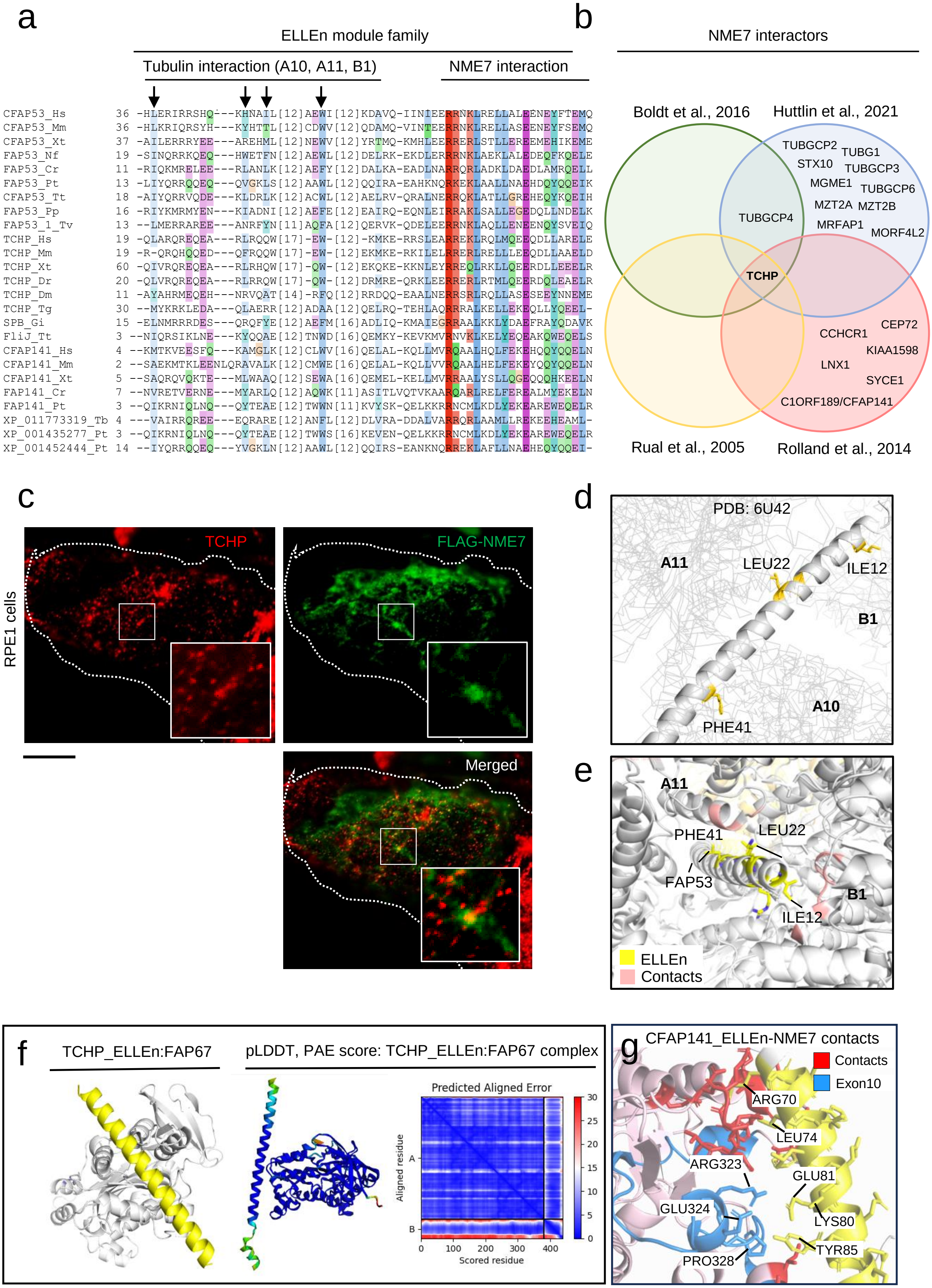

Supplementary Figure S8

a Expanded alignment of the ELLEn family proteins across species. Coloring schemes as per ClustalX parameters with modifications. Species represented are *Homo sapiens* (Hs), *Mus musculus* (Mm), *Xenopus tropicalis* (Xt), *Danio rerio* (Dr), *Drosophila melanogaster* (Dm), *Chlamodomonas reinhardtii* (Cr), *Physcomitrella patens* (Pp), *Tetrahymena thermophila* (Tt), *Trichomonas vaginalis* (Tv), *Naegleria fowleri* (Nf), *Paramecium tetraurelia* (Pt), *Toxoplasma gondii* (Tg), *Trypanosoma brucei* (Tb), and *Giardia lamblia* (Gl). **b** NME7 interactors as found in published interactome studies (37, 38, 40, 41). **c** Immunofluorescence microscopy images of hTERT-RPE-1 cells expressing FLAG-NME7. Cells were probed with antibodies against FLAG and TCHP. **d** Same structure as in Figure 8c showing more closely the conserved hydrophobic residues of ELLEn outermost N-terminus relative to the A and B tubule lattice. **e** The contact sites between the ELLEn outermost N-terminus residues and the outer doublet tubulin lattice. Isoleucine 12 makes contact to a protofilament B1 tubulin and Leucine 22 makes contact to a protofilament A11 tubulin. **f** Predicted complex by AlphaFold2 between the TCHP Ellen module (yellow) and FAP67 (grey). Right shows AlphaFold2 PAE and pLDDT scores of the complex. Models were generated in ColabFold (27) using the AlphaFold2 advanced suite. The N-terminal sequence portion bearing the ELLEn module from TCHP was extracted and combined with the full-length sequence of FAP67. Six recycles under sampling options was used to optimize the models and five models were generated. All five models recapitulated essentially the same complex structures for each. **g** Contacts between the ELLEn module of CFAP141 (yellow) and NME7 (PDB: 7RRO). NME7 contacts are shown in red and overlapping contacts corresponding gene product of *nme7* exon 10 are shown in blue. Source data are provided as a Source Data file.

Supplementary Figure S9

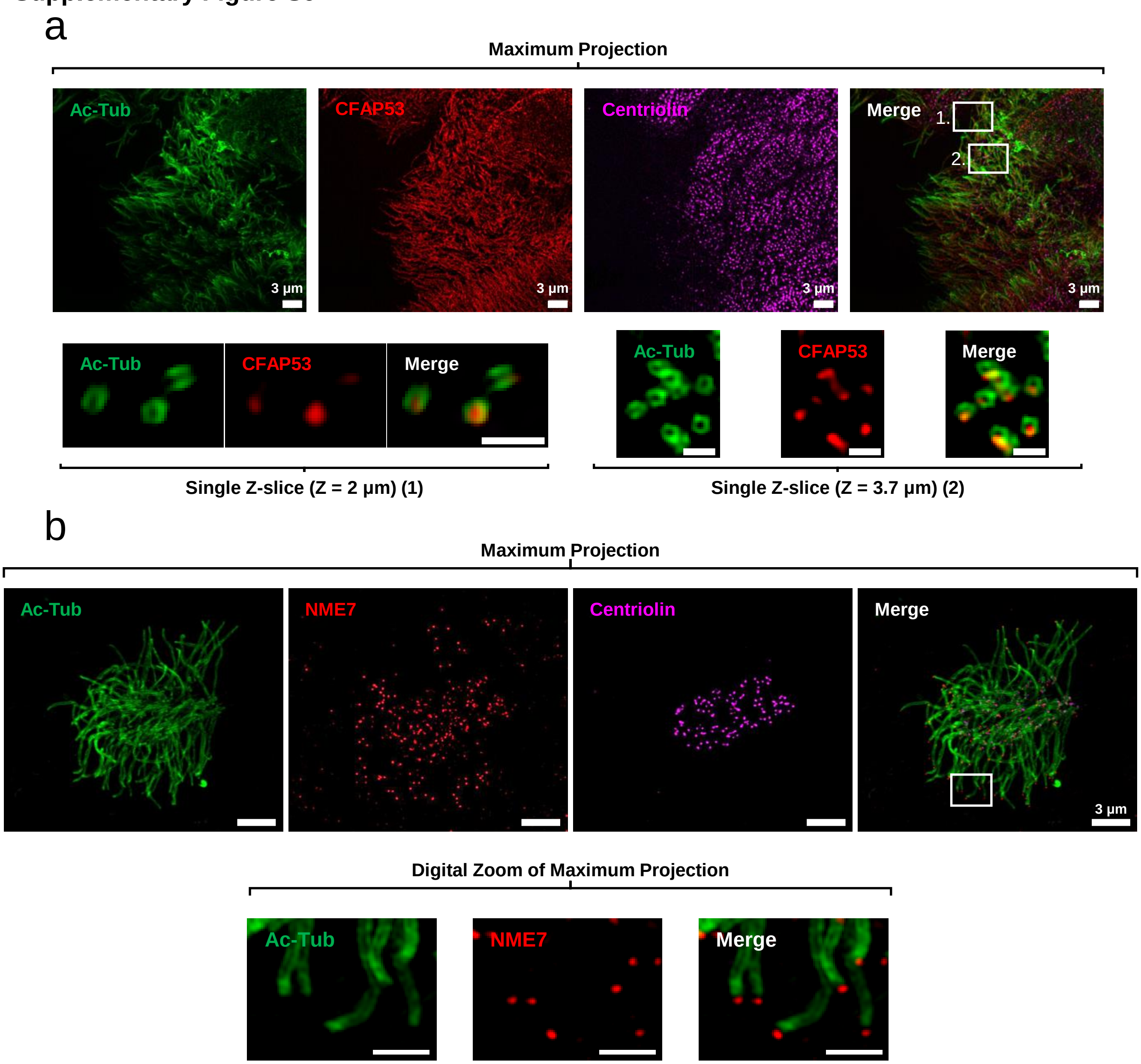

Supplementary Figure S9. Immunofluorescence microscopy micrographs of human **a** CFAP53 and **b** NME7 in motile cilia on cultured human bronchial epithelial cells imaged by structured illumination microscopy. Cells were stained with indicated antibodies. Images are representative of X=2 independent experiments.
