# Supplementary material for: Uncovering structural themes across cilia microtubule inner proteins with implications for human cilia function
Source: Nat Commun. 2024 Mar 27;15:2687. doi: 10.1038/s41467-024-46737-3 (PMC10973386; doi:10.1038/s41467-024-46737-3)
Supplement: Supplementary file 2 — Reporting Summary [file 41467_2024_46737_MOESM2_ESM.pdf]

nature portfolio

Corresponding author(s): Kenneth Bødtker Schou, Jens Skorstensgaard Andersen

Last updated by author(s): 20240223

Reporting Summary

Nature Portfolio wishes to improve the reproducibility of the work that we publish. This form provides structure for consistency and transparency in reporting. For further information on Nature Portfolio policies, see our Editorial Policies and the Editorial Policy Checklist.

Please do not complete any field with "not applicable" or n/a. Refer to the help text for what text to use if an item is not relevant to your study.
For final submission: please carefully check your responses for accuracy; you will not be able to make changes later.

Statistics

For all statistical analyses, confirm that the following items are present in the figure legend, table legend, main text, or Methods section.

n/a	Confirmed	
☐	☒	The exact sample size (*n*) for each experimental group/condition, given as a discrete number and unit of measurement
☐	☒	A statement on whether measurements were taken from distinct samples or whether the same sample was measured repeatedly
☐	☒	The statistical test(s) used AND whether they are one- or two-sided<br>*Only common tests should be described solely by name; describe more complex techniques in the Methods section.*
☒	☐	A description of all covariates tested
☒	☐	A description of any assumptions or corrections, such as tests of normality and adjustment for multiple comparisons
☒	☒	A full description of the statistical parameters including central tendency (e.g. means) or other basic estimates (e.g. regression coefficient) AND variation (e.g. standard deviation) or associated estimates of uncertainty (e.g. confidence intervals)
☒	☒	For null hypothesis testing, the test statistic (e.g. *F*, *t*, *r*) with confidence intervals, effect sizes, degrees of freedom and *P* value noted<br>*Give P values as exact values whenever suitable.*
☒	☐	For Bayesian analysis, information on the choice of priors and Markov chain Monte Carlo settings
☒	☐	For hierarchical and complex designs, identification of the appropriate level for tests and full reporting of outcomes
☒	☒	Estimates of effect sizes (e.g. Cohen's *d*, Pearson's *r*), indicating how they were calculated

Our web collection on statistics for biologists contains articles on many of the points above.

Software and code

Policy information about availability of computer code

Data collection	The profile-HMM searches were performed using publicly available databases i.e., the Uniclust30 (UniRef30_2023_02) for HHblits (HHsuite v3.3.0) searches and PfamA (pfamA_35.0) for the HHsearch (HHsuite v3.3.0).
Data analysis	Sequence remote homology searches were performed with HHsuite v3.3.0, HHpred (Version: 57c8707149031cc9f8edceba362c71a3762bbdf8), and GibbsCluster (version 2.0). Phylogenetic analyses were performed in IQ-Tree v.2.0. Protein intrinsical disorder was analysed in IUpred2a (PMID: 29860432) and DISOPRED (PMID: 15044227). Coiled-coil propensity was analyzed in HHpred (Version: 57c8707149031cc9f8edceba362c71a3762bbdf8). Alignments and ClustalW alignment analysis was performed in Jalview Version: 2.11.3.2. Protein structure analysis was performed in Pymol Version 2.5.5.<br>Code was deposited at https://github.com/Schoulab/MIP_structures/tree/main/Scripts DOI: 10.5281/zenodo.10683945.

Policy information about availability of data

All manuscripts must include a data availability statement. This statement should provide the following information, where applicable:

- Accession codes, unique identifiers, or web links for publicly available datasets
- A description of any restrictions on data availability
- For clinical datasets or third party data, please ensure that the statement adheres to our policy

Databases used: https://www.rcsb.org, https://www.cancer.gov/ccg/research/genome-sequencing/tcga, Uniclust30 (UniRef30_2023_02), and PfamA (pfamA_35.0).
Accessions codes: https://doi.org/10.2210/pdb8G2Z/pdb, https://doi.org/10.2210/pdb6U0H/pdb, https://doi.org/10.2210/pdb6U42/pdb, https://doi.org/10.2210/pdb8GLV/pdb, https://doi.org/10.2210/pdb8IYJ/pdb, https://doi.org/10.2210/pdb8SNB/pdb, https://doi.org/10.2210/pdb8J07/pdb, https://doi.org/10.2210/pdb7RRO/pdb.

Research involving human participants, their data, or biological material

Policy information about studies with human participants or human data. See also policy information about sex, gender (identity/presentation), and sexual orientation and race, ethnicity and racism.

Reporting on sex and gender	N/A
Reporting on race, ethnicity, or other socially relevant groupings	N/A
Population characteristics	N/A
Recruitment	N/A
Ethics oversight	N/A

Field-specific reporting

Please select the one below that is the best fit for your research. If you are not sure, read the appropriate sections before making your selection.

☒ Life sciences ☐ Behavioural & social sciences ☐ Ecological, evolutionary & environmental sciences

For a reference copy of the document with all sections, see nature.com/documents/nr-reporting-summary-flat.pdf

Life sciences study design

Sample size	For quantification of wt and CRISPR engineered cells, 200 cells per condition per experiment was counted. Micrographs were analyzed in Zeiss Zen 3.2. (blue edition) software. No sample size calculations were performed. Data for cilia formation and chromosome segregation errors were obtained by compiling representative images of cells followed by a visual assessment of cell staining relative to DAPI nuclear counting. A sample size of 200 was chosen based on the observed percentage change (in the order of 10-20%) between wildtype and NME7 KO cells, which was consistent among several NME7 KO clones compared to wildtype cells in an initial pilot test. The occurrence of these phenotypes was reproducible in three independent experiments, indicating that the phenotype strength permitted the chosen sample size.
Data exclusions	No data was excluded
Replication	Three independent experiments were performed and all attempts for replication were successful.
Randomization	The studies are performed on uniform cell cultures and hence we can assume that these cells are isogenic. In case of our CRISPR engineered NME7 KO cells, three different clones were tested and all three showed comparable phenotypes, minimizing the possibility of off-target effects.
Blinding	No blinding was applicable to these analysis. In case of NME7 KO hTERT-RPE-1 cells, the engineered cells showed noticeable differences in morphology and growth to the parental wildtype cells, which was identifiable before quantification. .

Behavioural & social sciences study design

All studies must disclose on these points even when the disclosure is negative.

Study description	
Research sample	
Sampling strategy	
Data collection	
Timing	
Data exclusions	
Non-participation	
Randomization	

Ecological, evolutionary & environmental sciences study design

All studies must disclose on these points even when the disclosure is negative.

Study description	
Research sample	
Sampling strategy	
Data collection	
Timing and spatial scale	
Data exclusions	
Reproducibility	
Randomization	
Blinding	

Did the study involve field work? ☐ Yes ☐ No

Field work, collection and transport

Field conditions	
Location	
Access & import/export	
Disturbance	

Reporting for specific materials, systems and methods

We require information from authors about some types of materials, experimental systems and methods used in many studies. Here, indicate whether each material, system or method listed is relevant to your study. If you are not sure if a list item applies to your research, read the appropriate section before selecting a response.

Materials & experimental systems

n/a	Involved in the study	
☐	☒	Antibodies
☐	☒	Eukaryotic cell lines
☒	☐	Palaeontology and archaeology
☒	☐	Animals and other organisms
☒	☐	Clinical data
☒	☐	Dual use research of concern
☒	☐	Plants

Methods

n/a	Involved in the study	
☒	☐	ChIP-seq
☐	☒	Flow cytometry
☒	☐	MRI-based neuroimaging

Antibodies

Antibodies used

Rabbit anti-TCHP (25931-1-AP, ProteinTech, no info on clone or lot number).
Rabbit anti-NME7 (NBP2-42888, Novus Biotechnology, no clone or lot number).
Rabbit anti-FLAG (PA1-984B, Invitrogen, RRID: AB_347227, no clone or lot number)
Mouse anti-FLAG M2 (F1804, Sigma, no clone or lot number),
Rabbit anti-CAPS2 (11924-1-AP, ProteinTech, no clone or lot number).
Rabbit anti-CAPS2 (HPA040004, Sigma, no clone or lot number).
Mouse anti-ARL13B (sc-515784, Santa Cruz, clone (C-5, no lot number)
Mouse anti-gamma-tubulin (MABT163, Sigma, clone 6H3.1, no lot number).
Mouse anti-Ac-tubulin (T7451, Sigma, clone 6-11B-1, no lot number).
Mouse anti-CNTRL (sc-365521), Santa Cruz, clone C-9, no lot number).
Rabbit anti-C2orf50 (HPA067681, Atlas Antibodies, lot: R97753, no clone number),
Rabbit anti-C11ORF1 (HPA038410, Sigma Aldrich, no clone or lot number).
Rabbit anti-GAPDH (#2118, CellSignal, clone 14C10, lot: 16).
Rabbit anti-Centrin 1 (12794-1-AP, ProteinTech, no clone or lot number).

Validation

Anti-FLAG (PA1-984B): Validation provided at https://www.thermofisher.com/antibody/product/DYKDDDDK-Tag-Antibody-Polyclonal/PA1-984B citing 41 studies confirming its specificity.
Rabbit anti-CAPS2 (11924-1-AP). Validation provided at https://www.ptglab.com/products/CAPS2-Antibody-11924-1-AP.htm . Antibody detects a protein of right size in WB of human placenta tissue lysates. Our WBs of hRPE1-TERT cell lysates detects a protein of similar size. No further validation is provided.
Anti-CAPS2 (HPA040004): Immunofluorescence microscopy
https://www.proteinatlas.org/ENSG00000180881-CAPS2/subcellular#human for different human three cell types. The centrosome staining is as also detected with the anti-CAPS2 (11924-1-AP (our study). No further validation exists.
Anti-ARL13B (sc-515784). Validation provided at https://www.scbt.com/p/arl13b-antibody-c-5 citing 9 peer reviewed studies confirming its specificity. Antibody detects a protein of right size in WB of human testis, human adrenal gland, and human eye tissue extracts.
Anti-gamma-tubulin (MABT163). Validation provided https://www.sigmaaldrich.com/DK/en/product/mm/mabt163 showing human IF staining and WB detection of a protein of right localization and size. No further validation exists.
Anti-Ac-tubulin (T7451). Validation provided at https://www.sigmaaldrich.com/DK/en/product/sigma/t7451?utm_source=google&utm_medium=cpc&utm_campaign=8939553962&utm_content=97228929004&gclid=CjwKCAiA_tuuBhAUEiwAvxkgTjhW4zdMk_xfbjcb9uyji8tn94Efhlgg_BOugleeP0566exmcVZhmRoCn9gQAvD_BwE citing 1077 peer reviewed studies confirming its specificity for human Ac-tubulin.
Anti-CNTRL (sc-365521). Validation provided at https://www.scbt.com/p/centriolin-antibody-c-9 citing 15 peer reviewed studies confirming its specificity. In addition, showing IF staining and WB detection of a human protein of right localization and size.
Anti-C2orf50 (HPA067681). Validation provided at https://www.proteinatlas.org/ENSG00000150873-C2orf50/tissue showing IF human tissue staining of bronchus and fallopian tube detecting motile cilia. No WB validation exist.
Anti-C11ORF1 (HPA038410). Validation provided at https://www.proteinatlas.org/ENSG00000137720-C11orf1/tissue showing IF human tissue staining of bronchus and fallopian tube detecting motile cilia. No WB validation exist.
Anti-GAPDH (#2118). Validation provided at https://www.cellsignal.com/products/primary-antibodies/gapdh-14c10-rabbit-mab/2118 citing 7799 peer reviewed studies. In addition, showing WB detection of a protein of the right size in humans.

Eukaryotic cell lines

Policy information about cell lines and Sex and Gender in Research

Cell line source(s)	The study uses Swiss NIH3T3 mouse fibroblasts (laboratory stock, originally derived from American Type Culture Collection (ATCC) clone CRL-1658) and human Retinal Pigment Epithelial 1 (hRPE1) cells, an hTERT RPE-1 cell line derived from a normal human retinal pigment epithelial (RPE) cell line, RPE-340, which was immortalized by the human telomerase reverse transcriptase subunit (hTERT). The hRPE1-hTERT cells were obtained from ATCC CRL-4000
Authentication	The hTERT RPE-1 cells used in the study were authenticated
Mycoplasma contamination	The hTERT RPE-1 cells used in the study were tested negative for mycoplasma contamination
Commonly misidentified lines (See ICLAC register)	No commonly misidentified cell lines were used in the study

Palaeontology and Archaeology

Specimen provenance	
Specimen deposition	
Dating methods	

☐ Tick this box to confirm that the raw and calibrated dates are available in the paper or in Supplementary Information.

Ethics oversight	

Note that full information on the approval of the study protocol must also be provided in the manuscript.

Animals and other research organisms

Policy information about studies involving animals; ARRIVE guidelines recommended for reporting animal research, and Sex and Gender in Research

Laboratory animals	
Wild animals	
Reporting on sex	
Field-collected samples	
Ethics oversight	

Note that full information on the approval of the study protocol must also be provided in the manuscript.

Clinical data

Policy information about clinical studies

All manuscripts should comply with the ICMJE guidelines for publication of clinical research and a completed CONSORT checklist must be included with all submissions.

Clinical trial registration	
Study protocol	
Data collection	
Outcomes	

Dual use research of concern

Policy information about dual use research of concern

Hazards

Could the accidental, deliberate or reckless misuse of agents or technologies generated in the work, or the application of information presented in the manuscript, pose a threat to:

No | Yes
- [x] No [ ] Yes — Public health
- [x] No [ ] Yes — National security
- [x] No [ ] Yes — Crops and/or livestock
- [x] No [ ] Yes — Ecosystems
- [x] No [ ] Yes — Any other significant area

Experiments of concern

Does the work involve any of these experiments of concern:

No | Yes
- [x] No [ ] Yes — Demonstrate how to render a vaccine ineffective
- [x] No [ ] Yes — Confer resistance to therapeutically useful antibiotics or antiviral agents
- [x] No [ ] Yes — Enhance the virulence of a pathogen or render a nonpathogen virulent
- [x] No [ ] Yes — Increase transmissibility of a pathogen
- [x] No [ ] Yes — Alter the host range of a pathogen
- [x] No [ ] Yes — Enable evasion of diagnostic/detection modalities
- [x] No [ ] Yes — Enable the weaponization of a biological agent or toxin
- [x] No [ ] Yes — Any other potentially harmful combination of experiments and agents

Plants

Seed stocks

Novel plant genotypes

Authentication

ChIP-seq

Data deposition

- [ ] Confirm that both raw and final processed data have been deposited in a public database such as GEO.
- [ ] Confirm that you have deposited or provided access to graph files (e.g. BED files) for the called peaks.

Data access links
May remain private before publication.

Files in database submission

Genome browser session
(e.g. UCSC)

Methodology

Replicates

Sequencing depth

Antibodies

Peak calling parameters

Data quality

Software

Flow Cytometry

Plots

Confirm that:

☒ The axis labels state the marker and fluorochrome used (e.g. CD4-FITC).

☒ The axis scales are clearly visible. Include numbers along axes only for bottom left plot of group (a 'group' is an analysis of identical markers).

☒ All plots are contour plots with outliers or pseudocolor plots.

☒ A numerical value for number of cells or percentage (with statistics) is provided.

Methodology

Sample preparation	RPE1 wt or RPE1/NME7 mutant cells were harvested by trypzination, fixed in ethanol (70%) and stained with PI supplemented with RNase A (10ug/ml) for 30 min at 37C
Instrument	BD FACSMelody Cell Sorter
Software	FlowJo™ v10.8 Software (BD Life Sciences)
Cell population abundance	100%
Gating strategy	We excluded debris and aggregates by gating on a forward scatter (FSC) vs. side scatter (SSC) plot. We applied a gate to include only single cells based on FSC-area vs. FSC-height.

☐ Tick this box to confirm that a figure exemplifying the gating strategy is provided in the Supplementary Information.

Magnetic resonance imaging

Experimental design

Design type	
Design specifications	
Behavioral performance measures	
Imaging type(s)	
Field strength	
Sequence & imaging parameters	
Area of acquisition	

Diffusion MRI ☐ Used ☐ Not used

Preprocessing

Preprocessing software	
Normalization	
Normalization template	
Noise and artifact removal	
Volume censoring	

Statistical modeling & inference

Model type and settings	
Effect(s) tested	

Specify type of analysis: ☐ Whole brain ☐ ROI-based ☐ Both

Statistic type for inference

(See Eklund et al. 2016)

Correction

Models & analysis

n/a	Involved in the study
☐	☐ Functional and/or effective connectivity
☐	☐ Graph analysis
☐	☐ Multivariate modeling or predictive analysis

Functional and/or effective connectivity

Graph analysis

Multivariate modeling and predictive analysis

This checklist template is licensed under a Creative Commons Attribution 4.0 International License, which permits use, sharing, adaptation, distribution and reproduction in any medium or format, as long as you give appropriate credit to the original author(s) and the source, provide a link to the Creative Commons license, and indicate if changes were made. The images or other third party material in this article are included in the article's Creative Commons license, unless indicated otherwise in a credit line to the material. If material is not included in the article's Creative Commons license and your intended use is not permitted by statutory regulation or exceeds the permitted use, you will need to obtain permission directly from the copyright holder. To view a copy of this license, visit http://creativecommons.org/licenses/by/4.0/
